# Incompatibility of the circadian protein BMAL1 and HNF4α in hepatocellular carcinoma

**DOI:** 10.1038/s41467-018-06648-6

**Published:** 2018-10-19

**Authors:** Baharan Fekry, Aleix Ribas-Latre, Corrine Baumgartner, Jonathan R. Deans, Christopher Kwok, Pooja Patel, Loning Fu, Rebecca Berdeaux, Kai Sun, Mikhail G. Kolonin, Sidney H. Wang, Seung-Hee Yoo, Frances M. Sladek, Kristin Eckel-Mahan

**Affiliations:** 1 Institute of Molecular Medicine, McGovern Medical School at the University of Texas Health Science Center (UT Health), Houston, TX 77030 USA; 2 Department of Molecular, Cell and Systems Biology, University of California Riverside, Riverside, CA 92521 USA; 3 Department of Pediatrics, Molecular and Cellular Biology, Children’s Nutrition Research Center, Baylor College of Medicine, Houston, TX 77030 USA; 4 Department of Integrative Biology and Pharmacology, McGovern Medical School at the University of Texas Health Science Center (UT Health), Houston, TX 77030 USA; 5 Department of Biochemistry and Molecular Biology, McGovern Medical School at the University of Texas Health Science Center (UT Health), Houston, TX 77030 USA

## Abstract

Hepatocyte nuclear factor 4 alpha (HNF4α) is a master regulator of liver-specific gene expression with potent tumor suppressor activity, yet many liver tumors express HNF4α. This study reveals that P1-HNF4α, the predominant isoform expressed in the adult liver, inhibits expression of tumor promoting genes in a circadian manner. In contrast, an additional isoform of HNF4α, driven by an alternative promoter (P2-HNF4α), is induced in HNF4α-positive human hepatocellular carcinoma (HCC). P2-HNF4α represses the circadian clock gene *ARNTL* (BMAL1), which is robustly expressed in healthy hepatocytes, and causes nuclear to cytoplasmic re-localization of P1-HNF4α. We reveal mechanisms underlying the incompatibility of BMAL1 and P2-HNF4α in HCC, and demonstrate that forced expression of BMAL1 in HNF4α-positive HCC prevents the growth of tumors in vivo. These data suggest that manipulation of the circadian clock in HNF4α-positive HCC could be a tractable strategy to inhibit tumor growth and progression in the liver.

## Abstract (web summary)

Hepatocyte nuclear factor 4 alpha (HNF4α) is regulated by different promoters to generate two isoforms, one of which functions as a tumor suppressor. Here, the authors reveal that induction of the alternative isoform in hepatocellular carcinoma inhibits the circadian clock by repressing BMAL1, and the reintroduction of BMAL1 prevents HCC tumor growth.

## Introduction

Hepatocellular carcinoma (HCC) is the leading hepatic malignancy found in humans and the second leading cause of all malignancy-related cancer deaths^1^. HCC is on the rise in the US and elsewhere, and has been linked to the increased incidence of nonalcoholic fatty liver disease, which is driven by the obesity epidemic^2^. Unfortunately, tumors are often found at a late stage with limited potential for surgical removal, making efforts to elucidate the mechanisms responsible for HCC tumor growth and metastasis paramount for improving patient prognosis.

The circadian clock is an intrinsic, 24-h time keeping system that operates in all cells of the body, governing rhythmicity in cell function including metabolism, gene expression, and trafficking and transport of cellular proteins^3–6^. Circadian disruption in humans has been linked to a number of diseases, including cancer^7–16^. Furthermore, experiments that mimic human jet-lag in mice reveal that circadian disruption is sufficient to induce spontaneous HCC^17^. The transcriptional activators, circadian locomoter output cycles kaput protein (CLOCK) and aryl hydrocarbon receptor nuclear translocator like (ARNTL, also known as BMAL1) form a heterodimer in hepatocytes and other cell types, and are necessary to drive the circadian transcription necessary for rhythmicity in many cellular events^6,18^.

Hepatocyte nuclear factor 4α (HNF4α) was originally identified as a nuclear factor enriched in the liver and important for control of genes involved in hepatocyte fate determination and function^19,20^. Since then, diverse roles for HNF4α have been described^16,21–26^, including its ability to function as a tumor suppressor, suppressing several genes (such as cyclin D1, *CCND1*) known to be causal for HCC initiation, growth, or progression^27–35^. While generally considered a transcriptional activator, several repressor proteins can also interact with HNF4α^22,36,37^, allowing repression of some target genes. Some evidence suggests that human HCC may be deficient in HNF4α expression^32^, and overexpression of one of its isoforms in mice reverses the growth of liver tumors arising from chemically induced mutations^32^, while depletion of this isoform promotes tumor growth^38^.

Complicating the mechanistic understanding of HNF4α in liver cancer is the fact that there are two promoters (P1 and P2) that drive the expression of different *HNF4a* transcript variants, which are differentially expressed not just in human HCC, but also colon cancer^28,39,40^. The P1 promoter gives rise to HNF4α1/2 which is expressed in normal adult liver, while the P2 promoter gives rise to HNF4α7/8, which is not normally expressed in the adult liver, but is in fetal liver as well as HCC^39,41^. While P1-HNF4α is typically found only in the nucleus, posttranslational modifications can promote cytoplasmic trafficking^40,42,43^.

Our results reveal that the two isoforms of HNF4α (“P1-HNF4α” and “P2-HNF4α”), which are differentially expressed in liver cancer, exhibit distinct circadian roles. While P1-HNF4α normally represses cell cycle and epithelial-to-mesenchymal transition (EMT) genes in a circadian manner, P2-HNF4α is selectively induced in HCC, where it directly inhibits the expression of the circadian protein BMAL1 and leads to the cytoplasmic expression of the P1 isoform. Importantly, forced expression of BMAL1 in HNF4α-positive liver cancer cells impairs spheroid growth in culture and tumor growth in vivo, demonstrating that manipulation of the circadian clock in HNF4α-positive HCC could be a realistic strategy to slow or reverse growth of human HCC.

## Results

### HNF4α is heterogeneously expressed in human HCC

**Fig. 1:**
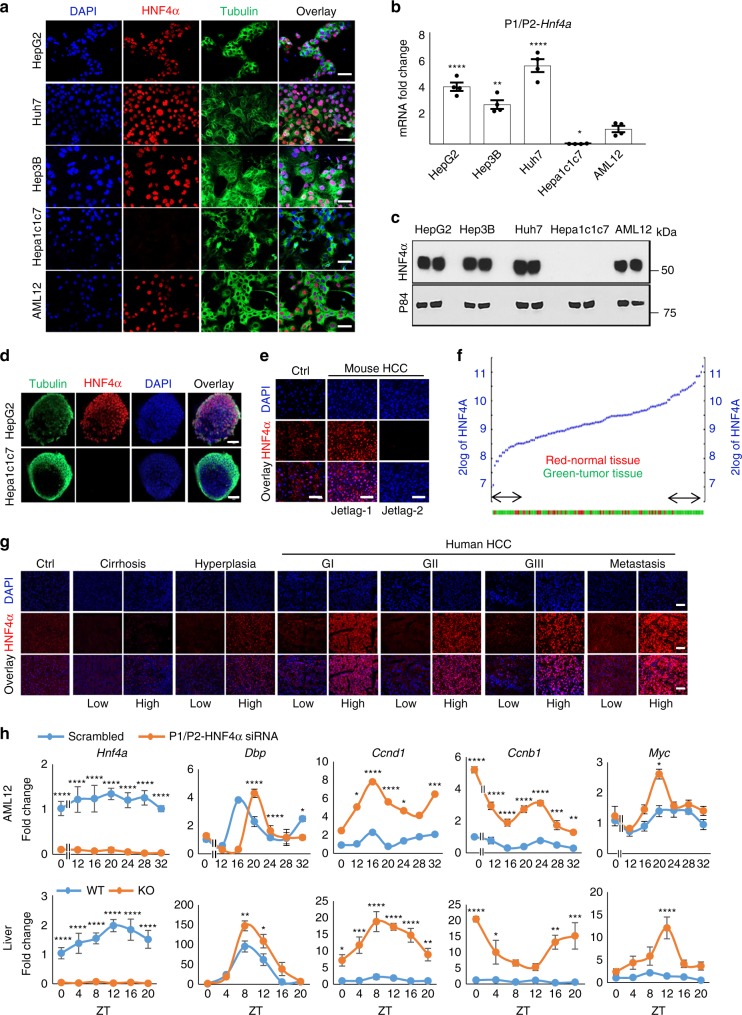
HNF4α is heterogeneously expressed in HCC. **a** Immunofluorescence (IF) reveals P1/P2-HNF4α expression and subcellular localization in mouse and human HCC, hepatoblastoma cancer lines, and in the nontransformed liver cell line, AML12, grown in monolayer conditions. **b** RT-PCR reveals mRNA abundance of P1/P2-HNF4α in HCC cell lines in vitro, fold change over AML12 *Hnf4a* mRNA. Compared to ZT0 at the same time, ^*^P < 0.01, ^**^P < 0.01, ^***^P < 0.001, ^****^P < 0.0001, one-way ANOVA test, Dunnett’s multiple comparisons test. (*N* = 4). **c** Western blot showing P1/P2-HNF4α abundance in whole cell lysates of cancer cell lines and nontransformed AML12 cells. **d** Staining of 3D, HCC- and hepatoblastoma-derived spheroids from HepG2 and Hepa-1c1c7 cells with antibodies against P1/P2-HNF4α. **e** IF staining of spontaneous HCC isolated from jet-lagged WT mice using antibody to P1/P2-HNF4α. **f** mRNA expression of *HNF4a* in human HCC tumors and surrounding normal liver tissue (R2: genomics analysis and visualization platform). **g** IF staining of human normal (“Ctrl”), cirrhotic, hyperplastic, and HCC tumor tissue using antibody against P1/P2-HNF4α (G = grades, advancement increasing with number). **h** RT-PCR reveals the circadian expression of *P1/P2*-*Hnf4a*, *Dbp, Ccnd1*, *Ccnb1* and *Myc* following the application of scrambled (Sc) or siRNA specific to *P1/P2-Hnf4a* in AML12 cells (top panel) or in the liver of WT and HNF4α knockout (KO) mice (bottom panel). Compared to controls at the same time, ^*^P < 0.03, ^**^P < 0.005, ^***^P < 0.001, ^****^P < 0.0001, two-way ANOVA test, Sidak’s multiple comparisons test. (*N* = 8–43). Scale bar is 50 µm. (See Supplementary Table 1 for JTK_Cycle Rhythmicity Statistics.) Error bars = SEM

While evidence suggests that HNF4α has tumor suppressive effects in the liver^38^, heterogeneity of HNF4α expression in HCC has largely been observed using antibodies that do not distinguish between the P1 and P2 isoforms. To reassess HNF4α heterogeneity in liver cancer, mouse and patient-derived human HCC and hepatoblastoma cell lines were first stained using an antibody recognizing both isoforms (P1 and P2) of HNF4α (Fig. 1a). Several HCC cell lines expressed P1/P2-HNF4α robustly while Hepa-1c1c7 cells lacked HNF4α. The nontransformed hepatocyte-derived AML12 cell line also expressed P1/P2-HNF4α, as did the human cancer line HepG2, which is commonly used as an in vitro model for HCC, but is more appropriately classified as hepatoblastoma^44,45^ (Fig. 1a). Using PCR primers and immunoblotting reagents that recognize both the P1 and P2 isoforms, similar patterns were observed: Hepa-1c1c7 cells were devoid of P1/P2 transcripts and proteins, while AML12, HepG2, Huh7 and Hep3B cells all expressed *HNF4a* mRNA and protein (Fig. 1b, c). Because cells grown in two-dimensional (2D) culture do not always retain normal patterns of gene expression (reviewed in^46^), we cultured HNF4α-positive HepG2 cells and HNF4α-negative Hepa-1c1c7 cells in Matrigel to generate small 3D spheroids. HepG2 spheroids stained with an antibody recognizing both isoforms of HNF4α showed robust HNF4α expression while Hepa-1c1c7-derived spheroids were devoid of the protein (Fig. 1d). These results indicate that 2D vs. 3D growth conditions alone did not account for the presence or lack of HNF4α.

To expand our understanding of HNF4α heterogeneity in HCC, spontaneous HCC isolated from mice subjected to a protocol that simulates jet-lag in humans (so called “jet-lagged” mice)^17^ were stained for P1/P2-HNF4α protein. Heterogeneity in HNF4α expression was also observed in HCC specimens from jet-lagged mice (Fig. 1e). Furthermore, analysis of microarray data from human HCC (R2: Genomics Analysis and Visualization Platform, http://hgserver1.amc.nl/cgi-bin/r2/main.cgi) revealed heterogeneity in *HNF4a* transcript, with HCC displaying extremely high or extremely low levels of *HNF4a* mRNA compared to control tissue (Fig. 1f). Staining for P1/P2-HNF4α protein in human HCC arrays revealed that approximately half of HCC tumors are positive for P1/P2-HNF4α (Fig. 1g and Supplementary Fig. 1a). While HCC is more common in males than females^47^, the heterogeneity in HNF4α expression was not sex-specific. HNF4α-positive tumors analyzed for intensity across grades, revealed that metastatic regions expressed higher levels of P1/P2-HNF4α compared to grades 1–3 or to cirrhotic and hyperplastic regions (Fig. 1g and Supplementary Fig. 1b). Thus, while nontransformed liver cells and control tissue express moderate levels of HNF4α, HCC tumors are heterogeneous in their expression of HNF4α, and HNF4α-positive tumors have significantly higher expression of the protein compared to normal liver (Fig. 1g and Supplementary Fig. 1b).

### HNF4α has circadian activity in liver cells

Based on studies suggesting that HNF4α may influence the liver circadian clock^23^, we examined whether a circadian function of HNF4α might differ in the context of HNF4α-positive HCC by evaluating target gene expression. Primary mouse hepatocytes and AML12 cells were serum shocked to synchronize the circadian clock across the cells. The expected circadian nuclear translocation of BMAL1 was observed in primary mouse hepatocytes following serum shock (Supplementary Fig. 1c) and *Dbp* (a canonical CLOCK:BMAL1 target gene) showed the expected circadian oscillation (Supplementary Fig. 1d, *P* = 0.0199). To assess whether HCC and hepatoblastoma lines were able to synchronize in culture, Hepa-1c1c7, Hep3B, and HepG2 cells were serum shocked and *Dbp* expression was examined. The cancer cells were also able to synchronize and sustain 24-h rhythmicity, as indicated by oscillatory *Dbp* expression in Hepa-1c1c and Hep3B cells (*P* = 0.027 and 0.004, respectively, JTK_Cycle test for rhythmicity^48^, Supplementary Fig. 1d and Supplementary Table 1). To determine whether loss of HNF4α altered the circadian expression of target genes, we treated AML12 cells with siRNA against both P1/P2 isoforms of *Hnf4a*. In addition, we inducibly knocked out HNF4α in the adult mouse liver using a previously described mouse model^27^. Reducing P1/P2-HNF4α expression in AML12 cells and healthy liver did not eliminate rhythmicity of the core clock genes, though *Dbp* was phase shifted in AML12 cells (Fig. 1h, Supplementary Fig. 1e, f, and Supplementary Table 1). In contrast, *Myc* expression was significantly increased in AML12 cells in the absence of P1/P2-HNF4α, and the HNF4α target genes, cyclin B1 (*Ccnb1*) and cyclin D1 (*Ccnd1*), which P1-HNF4α has been shown to repress at single time points^27^, showed robust rhythmicity in expression in the HNF4α KO liver (*P* = 0.0026 and *P* = 0.0075, respectively) (Fig. 1h and Supplementary Table 1), and an increase at the protein level (Supplementary Fig. 1g, h). Though the mechanisms are not known, HNF4α transcriptionally inhibited target genes at different phases. Being one of the most abundant hepatic transcription factors, HNF4α binds to over 20,000 locations in the genome^49^, and plausibly may activate or repress at different *zeitgeber* times based on the temporal array of different cofactors shared. Loss of P1/P2-HNF4α had no significant impact on the circadian expression of regulators of EMT, such as Snail (*Snai1*), Slug (*Snai2*), and E-cadherin (*Cdh1*), in either AML12 cells or the liver (Supplementary Fig. 1e, f and see Supplementary Table 1). To determine whether circadian de-repression of HNF4α target genes was BMAL1-dependent, we used siRNA to knockdown BMAL1 in the presence or absence of siRNA to *P1/P2-Hnf4a* and analyzed *Ccnd1* expression following serum shock in AML12 cells (Supplementary Fig.1i). Interestingly, while loss of BMAL1 or P1/P2-HNF4α both increased *Ccnd1* expression, co-treatment did not further augment the effect (Supplementary Fig. 1j). Thus, nontransformed cells as well as HNF4α-positive and -negative HCC cells can exhibit 24-h rhythmicity, and in nontransformed liver cells, P1/P2-HNF4α represses the circadian expression of *Myc*, *Ccnb1*, and *Ccnd1*, which are involved in cell growth and proliferation.

### Loss of HNF4α and BMAL1 co-expression in HCC

**Fig. 2:**
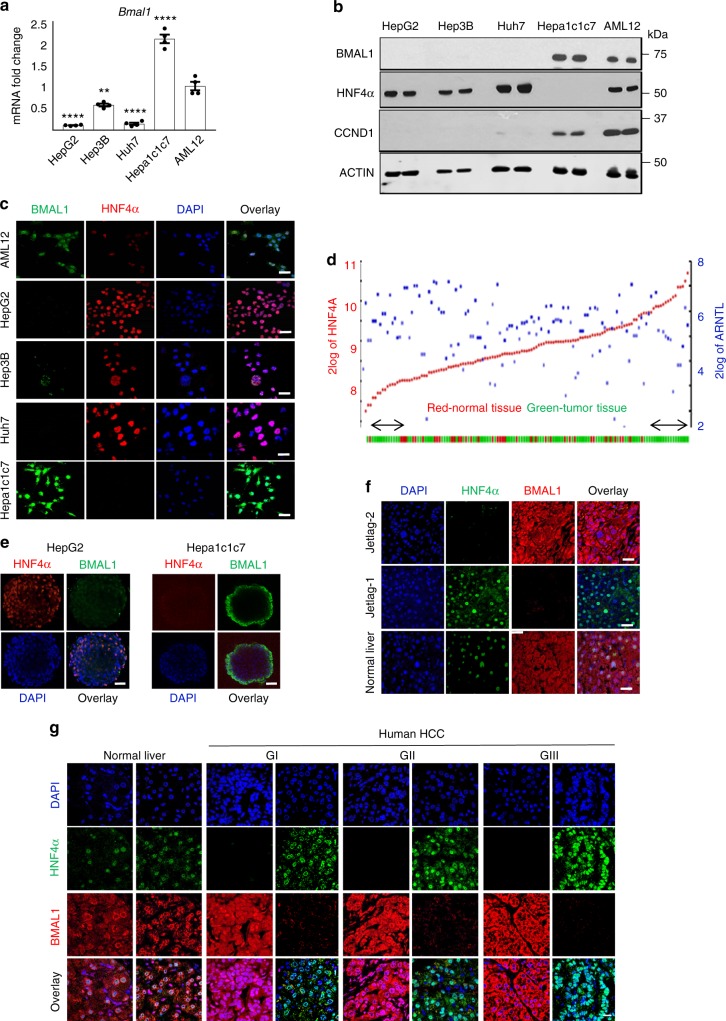
Inverse expression of HNF4α and BMAL1 in HCC. **a** RT-PCR reveals *ARNTL* (*BMAL1*) mRNA abundance in HCC and hepatoblastoma lines expressing varying levels of *P1/P2*-*Hnf4a* mRNA as well as the nontransformed hepatocyte cell line, AML12. Levels of *BMAL1* mRNA expression compared to AML12 cells, ^**^P < 0.001, ^****^P < 0.0001, one-way ANOVA test, Dunnett’s multiple comparisons test. (*N* = 4*)*. **b** Western blot reveals BMAL1, P1/P2-HNF4α, and CCND1 protein levels in HNF4α-positive and HNF4α-negative liver cancer lines as well as in nontransformed AML12 cells. **c** Staining of 2D HCC cells and AML12 cells with antibodies to BMAL1 and to P1/P2-HNF4α. Overlap with DAPI nuclear stain. **d** Human HCC microarray datasets reveal inverse gene expression of *HNF4a* and *ARNTL* (*BMAL1*) mRNA in HCC specimens (*P* < 0.0017) (*N* = 134). **e** Staining of 3D spheroids generated from HepG2 and Hepa-1c1c7 cells with antibody to P1/P2-HNF4α and BMAL1. Overlay with DAPI nuclear signal. **f** Staining of spontaneous mouse HCC from jet-lagged mice using antibodies to BMAL1 and P1/P2-HNF4α. Overlay with DAPI nuclear stain. **g** Human HCC specimens stained with antibodies for BMAL1 and P1/P2-HNF4α. Overlay with DAPI nuclear stain (G = tumor grade, increasing with number). Scale bar is 50 µm. Error bars = SEM

While BMAL1 and HNF4α are co-expressed in healthy hepatocytes and nontransformed hepatocyte-derived cell lines (Fig. 2a–c, Supplementary Fig. 1a, g, h), we found that cancer cells expressing P1/P2-HNF4α express little to no BMAL1. On the other hand, HCC that is devoid of HNF4α expresses high levels of BMAL1 mRNA and protein (Fig. 2a–c). Analysis of human HCC microarray data sets (R2: Genomics Analysis and Visualization Platform), revealed that *ARNTL* (BMAL1) and *HNF4A* are also inversely correlated at the level of mRNA expression in human HCC (*P* < 0.0017; *N* = 134) (Fig. 2d). Both 2D and 3D culture of HepG2 cells confirmed that while the HNF4α-positive HepG2-cells did not express BMAL1 (Supplementary Fig. 2a), HNF4α–deficient Hepa-1c1c7 spheroids robustly expressed Bmal1 (Fig. 2b, c and Supplementary Fig. 2b). HepG2 spheroids infected with a lentiviral construct for BMAL1 showed reduced HNF4α expression (Supplementary Fig. 2a), while Hepa-1c1c7 spheroids infected with an HNF4α-containing vector showed reduced BMAL1 expression in synchronized organoid cultures (Supplementary Fig. 2b), suggesting an inverse relationship between BMAL1 and HNF4α in liver cancer cells. Similarly, GFP-sorted HepG2 cells expressing GFP-BMAL1 showed reduced HNF4α, while GFP-sorted Hepa-1c1c7 cells expressing GFP-HNF4α showed reduced *Bmal1* mRNA abundance, confirming an inverse correlation between HNF4α and BMAL1 in the context of HCC (Supplementary Fig. 2c, d). To determine whether this apparent incompatibility of expression was present in spontaneous HCC, HCC from jet-lagged mice were stained with antibodies for BMAL1 and P1/P2-HNF4α. Spontaneous HCC from jet-lagged mice also revealed that high HNF4α expression coincided with low BMAL1 expression and vice versa (Fig. 2f). Finally, human HCC sections stained with antibodies to P1/P2-HNF4α and BMAL1 revealed the same inverse relationship (Fig. 2g and Supplementary Fig. 2e).

### P2-HNF4α is induced in HCC and has unique circadian activity

**Fig. 3:**
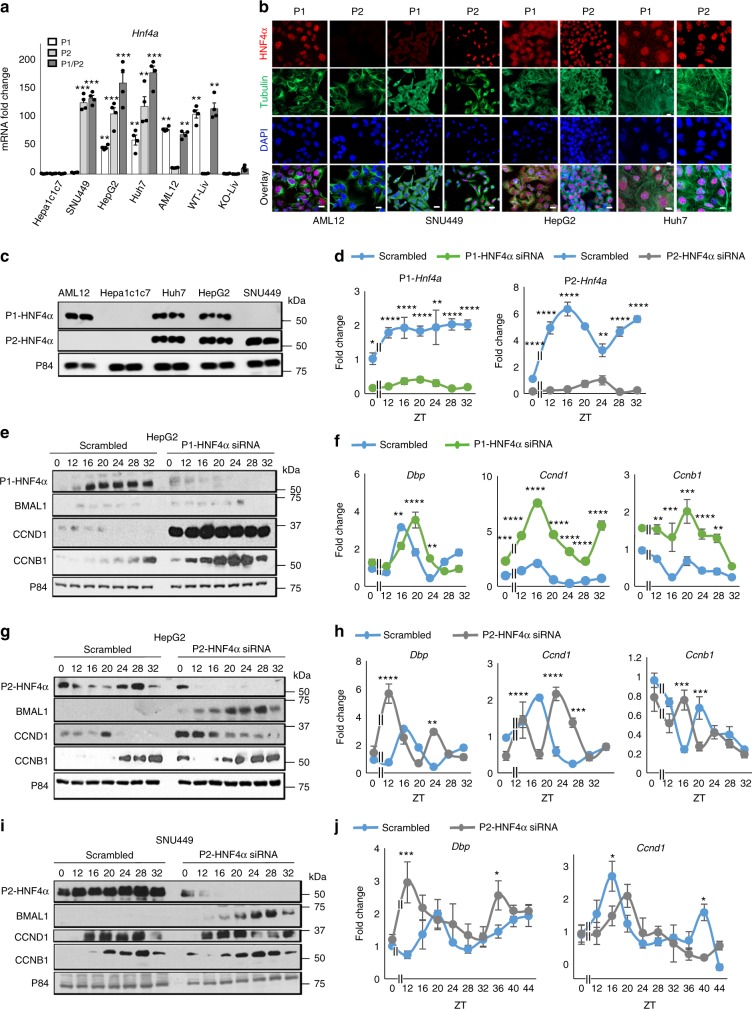
The P2 isoform of HNF4α is uniquely expressed in HCC and has distinct circadian activity. **a** RT-PCR reveals the mRNA abundance of *P1-HNF4a* and *P2-HNF4a* in hepatoblastoma and HCC lines, nontransformed AML12 cells, and wild-type (WT) and *Hnf4a* knockout (KO) liver tissues using primers to each isoform. **b** Staining of AML12, hepatoblastoma, and HCC cell lines with antibodies specific to P1-HNF4α or P2-HNF4α. Overlay with DAPI nuclear stain. **c** Western blot of lysates from AML12, hepatoblastoma, and HCC cell lines with antibodies specific to P1-HNF4α, P2-HNF4α, or P84 proteins. **d** RT-PCR reveals expression of *P1-HNF4a* or *P2-HNF4a* following serum synchronization in HepG2 cells and using siRNA specific to *P1-HNF4a* (left panel) or *P2*-*HNF4a* (right panel). **e** Western blot analysis showing P1-HNF4α, BMAL1, CCND1, and CCNB1 protein expression in HepG2 cells serum shocked and previously treated with siRNA specific to *P1*-*HNF4a* or with scrambled oligonucleotides. **f** RT-PCR reveals the expression of *DBP*, *CCND1*, and *CCNB1* following serum shock and knockdown of *P1*-*HNF4a* with specific siRNA or scrambled (Sc) oligonucleotides. **g** Western blot showing P2-HNF4α, BMAL1, CCND1, and CCNB1 protein expression following expression of scrambled or *P2-HNF4α*-specific siRNA oligonucleotides. **h** RT-PCR reveals the circadian expression of *DBP*, *CCND1*, and *CCNB1* following knockdown of P2-HNF4α. **i** Western blot showing P2-HNF4α, BMAL1, CCND1, and CCNB1 expression in SNU449 cells after serum shock and previously treated with scrambled oligonucleotides or siRNA oligonucleotides specific to P2-HNF4α. **j** RT-PCR reveals the expression of *DBP* and *CCND1* following the application of scrambled (Sc) or siRNA specific to the *P2-HNF4a* isoform in SNU449 cells. Two-way ANOVA, Sidak’s multiple comparisons test, ^*^P < 0.03, ^**^P < 0.005, ^***^P < 0.0005, ^****^P < 0.0001. (*N* = 4). Scale bar 50 µm. (See Supplementary Table 1 for JTK_Cycle Rhythmicity Statistics.) Error bars = SEM

While the P1 promoter-driven isoform is mainly expressed in the liver of adult mice, both P1-HNF4α and P2-HNF4α are expressed in the normal gut^39,40,50^ (Supplementary Fig. 2F), where only the P1-HNF4α has tumor suppressive activity and aberrant expression of P2-HNF4α contributes to colitis-associated colon cancer^28,30^. P2-HNF4α is also found in HCC^39^, and has recently been associated with poor prognosis^51^. Using primers detecting only P1-HNF4α or P2-HNF4α we analyzed the expression of each isoform across normal and transformed liver cells. While Hepa-1c1c7 had no detectable P1- or P2-HNF4α, SNU449, HepG2, Hep3B, and Huh7 cells expressed both P1- and P2-HNF4α or only P2-HNF4 (SNU449). P2- HNF4α was not detectable in normal liver tissue (Fig. 3a). Antibodies specific to either P1-HNF4α or P2-HNF4α revealed that AML12 cells also do not express detectable P2-HNF4α, while all the HNF4α-positive cancer lines do (Fig. 3b, c). To determine whether P1-HNF4α is indeed responsible for the circadian transcriptional repression observed in Fig. 1h, HNF4α isoform-specific siRNA was administered to HepG2 cells. In control cells, robust oscillation of *P2-HNF4*a was observed (*P* = 0.0008), compared to *P1-HNF4a*, which did not oscillate (Fig. 3d and Supplementary Table 1). Loss of both P1- and P2-HNF4α resulted in a pronounced upregulation of BMAL1, CCND1, and CCNB1 proteins and an upregulation of the BMAL1 target *DBP*, as well as circadian induction of *CCND1* and *CCNB1* transcripts after serum synchronization (Supplementary Fig. 3a, b). Interestingly, knockdown of only the P1 isoform of HNF4α did not affect BMAL1 abundance, while CCND1 and CCNB1 were greatly induced and *CCND1* exhibited rhythmicity at the level of mRNA (*P* = 6.80E^−^^05^) (Fig. 3e, f). Overexpression of the P1 isoform in HNF4α-deficient Hepa-1c1c7 cells resulted in the inverse outcome, with the levels of CCND1 and CCNB1 being reduced at the mRNA and protein levels (Supplementary Fig. 3E). Remarkably, knockdown of only the P2 isoform resulted in a robust increase in BMAL1 protein, but no overall increase in cyclin gene expression, although there were differences at individual time points (Fig. 3g, h). Importantly, the loss of P1- or P2-HNF4α did not significantly affect the gene expression of the alternate isoform (Supplementary Fig. 3C). Knockdown of P2-HNF4α in SNU449, a human HCC line that exhibits only P2 expression, resulted in a similarly robust increase in BMAL1, while CCND1 was minimally affected (Fig. 3i, j). Together, these data indicate that the repressive effect of P1-HNF4α on cell cycle genes is circadian, and ectopic expression of P1-HNF4α can induce circadian transcriptional repression in normal and HCC cells. In contrast, P2-HNF4α reduces the expression of the circadian protein BMAL1 in HCC.

### Circadian control of migration and invasion by HNF4α

**Fig. 4:**
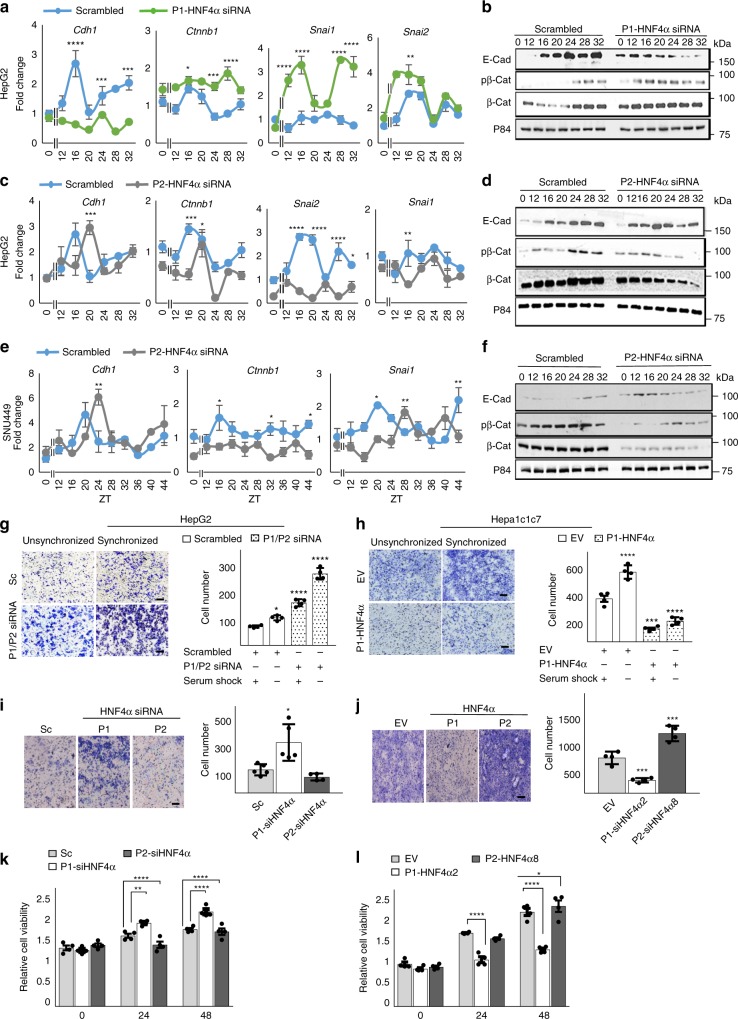
Circadian control of EMT by HNF4α is isoform specific. **a** RT-PCR reveals mRNA abundance of EMT genes *CDH1, CTNNB1, SNAI1*, and *SNAI2* after serum shock with prior application of scrambled oligonucleotides or siRNA specific to P1-HNF4α in HepG2 cells. **b** Western blot showing expression of CDH1, phosphorylated, and total β-catenin (CTNNB1, β-Cat), after P1-HNF4α knockdown followed by serum shock. **c** RT-PCR reveals expression of EMT genes following serum shock and prior application of scrambled or siRNA specific to *P2-HNF4a*. **d** Western blot showing expression of CDH1, phosphorylated and total β-Cat following knockdown of P2-HNF4α and serum shock. **e** RT-PCR reveals circadian expression of EMT genes *CDH1*, *CTNNB1*, and *SNAI1* after inhibition of P2-HNF4α using specific siRNA oligonucleotides in SNU449 cells. **f** Western blot showing the expression of CDH1 and phosphorylated and total β-Cat after P2-HNF4α knockdown following serum shock in SNU449 cells. Two-way ANOVA, Sidak’s multiple comparisons test, ^*^P < 0.03, ^**^P < 0.005, ^***^P < 0.0005, ^****^P < 0.0001, (*N* = 4). **g** Invasion assay reveals invaded unsynchronized or circadian synchronized HepG2 cells expressing scrambled or siRNA for *P1/P2-HNF4a*, 48 h after plating. Quantification, right panel. **h** Invaded unsynchronized or synchronized Hepa-1c1c7 cells following serum synchronization and prior overexpression of P1-HNF4α. Quantification, right panel. **i** Invaded synchronized HepG2 cells following application of scrambled (“Sc”), *P1-HNF4a*, or *P2-HNF4a* siRNA oligonucleotides. Quantification, right panel. **j** Invaded synchronized Hepa-1c1c7 cells following overexpression of P1-HNF4α or P2-HNF4α. Quantification, right panel. Compared to SC or EV at the same time: ^*^P < 0.05, ^**^P < 0.01, ^***^P < 0.001, ^****^P < 0.0001, one-way ANOVA test, Dunnett’s multiple comparisons test. (*N* = 5). **k** Fold change in proliferating HepG2 cells following P1-HNF4α vs. P2-HNF4α knockdown at 24- and 48 h using MTT assay. **l** MTT assay reveals proliferating Hepa-1c1c7 cells after transfection with empty vector (EV), *P1-Hnf4a* (*Hnf4a2*) or *P2-Hnf4a* (*Hnf4a8*) at 24- and 48 h using MTT assay. Comparing SC/EV to P1/P2-siHNF4α or between P1-siHNF4α and P2-siHNF4α: Two-way ANOVA, Sidak’s multiple comparisons test, ^*^P < 0.03, ^**^P < 0.005, ^***^P < 0.0005, ^****^P < 0.0001, (*N* = 6). Scale bar 100 µm. (See Supplementary Table 1 for JTK_Cycle Rhythmicity Statistics.) Error bars = SEM

Part of P1-HNF4α’s tumor suppressor activity has been attributed to its ability to repress genes involved in EMT^32,52^. To determine whether P1-HNF4α or P2-HNF4α differentially regulate EMT in transformed cells, single or double knockdown of P1-HNF4α and P2-HNF4α was performed in HepG2 cells prior to serum shock using siRNA. In contrast to AML12 cells, knockout of both P1-HNF4α and P2-HNF4α concomitantly in HepG2 cells resulted in depression of CDH1 expression, but activation of β-catenin *(CTNNB1), SNAI1*, and *SNAI2* (Supplementary Fig. 4b, c). Similarly, CDH1 was reduced following P1/P2-HNF4α inhibition while both phospho- and total β-catenin were increased (Supplementary Fig. 4b, c). Knockdown of only P1-HNF4α in HepG2 cells largely mimicked this result, with *CTNNB1*, *SNAI2*, and *SNAI1* mRNAs being increased in abundance throughout the circadian cycle, while CDH1 expression was reduced at both the mRNA and protein levels (Fig. 4a, b). Loss of P1-HNF4α in HepG2 cells also resulted in an increase β-catenin phosphorylation (Fig. 4b), while overexpression of P1-HNF4α in Hepa-1c1c7 cells had the opposite effect (Supplementary Fig. 4a, b). In contrast, knockdown of P2-HNF4α in HepG2 and SNU449 cells revealed that P2-HNF4α has differential circadian effects on EMT genes compared to the P1 isoform. For example, the mRNA and protein abundance of CDH1 was increased at specific time points in the absence of P2-HNF4α (Fig. 4c, e), while CTNNB1 protein was moderately decreased (Fig. 4d, f). Finally, in contrast to the loss of P1-HNF4α, knockdown of P2-HNF4α reduced *SNAI1* and *SNAI2* mRNA (Fig. 4c, e, and Supplementary Fig. 4f).

To determine whether loss of P1-HNF4α vs. P2-HNF4α differentially affected EMT, HepG2 cells were infected with siRNA to both or single HNF4α isoforms prior to circadian synchronization and invasion assays, wherein the ability of cells to migrate through Matrigel toward a chemoattractant was measured. Knockdown of P1/P2-HNF4α resulted in an increase in the invasiveness of HepG2 cells (Fig. 4g), congruent with previously published results showing that P1 overexpression in cells reduces invasion^32^. Circadian synchronization positively affected invasion, though the reasons for this are unclear. Ectopic BMAL1 expression in HepG2 cells decreased their invasive potential, which was largely recovered by concomitant knockdown of P1/P2-HNF4α (Supplementary Fig. 4g). Overexpression of P1-HNF4α in HNF4α-deficient Hepa-1c1c7 cells produced an inverse phenotype, with cells invading less in both unsynchronized and synchronized conditions (Fig. 4h). When HepG2 cells were transfected with siRNA to only P1-HNF4α or P2-HNF4α, invasiveness of the cells was only increased after losing expression of the P1 isoform (Fig. 4i), and P1-HNF4α vs. P2-HNF4α overexpression in HNF4α-deficient Hepa-1c1c7 cells revealed a loss of invasion only after P1-HNF4α but not P2-HNF4α overexpression (Fig. 4j).

To determine whether HNF4α isoform-specific expression differentially affects cell proliferation, MTT assays (colorimetric assays designed to assess cell viability) were performed on HepG2 cells lacking both P1/P2 (Supplementary Fig. 4h) or each individual isoform (Fig. 4k and Supplementary Fig. 4i), or on Hepa-1c1c7 overexpressing one or the other isoform (Fig. 4l and Supplementary 4j). Knockdown of both isoforms resulted in an increase in HepG2 cell proliferation at 24- and 48 h (Supplementary Fig. 4h), which was due to the loss of the P1 isoform, as demonstrated by application of isoform-specific siRNAs (Fig. 4k). Overexpression of P1-HNF4α reduced the number of viable Hepa-1c1c7 cells at both time points, while overexpression of P2 had no effect (Fig. 4l). Thus, P1-HNF4α functions as a repressor of EMT and cell proliferation in HCC, but P2-HNF4α does not.

### The P1 isoform of HNF4α is aberrantly localized in HCC

**Fig. 5:**
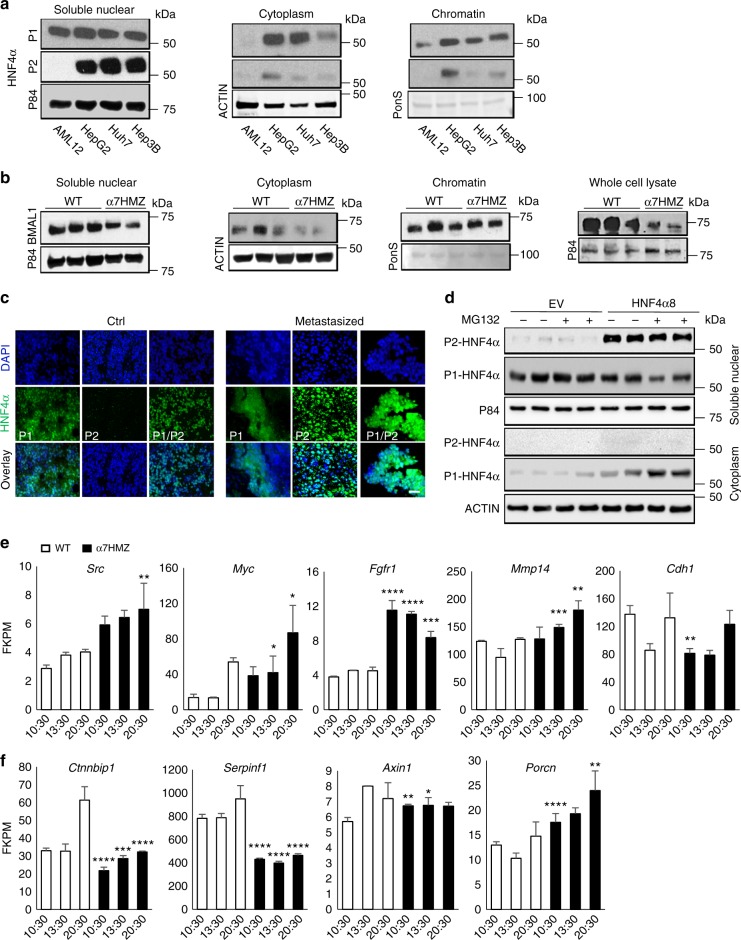
Altered subcellular localization of HNF4α isoforms in HCC. **a** Western blot shows the expression of P1-HNF4α and P2-HNF4α in the soluble nuclear, cytoplasmic, and chromatin fractions of AML12, hepatoblastoma, and HCC lines. **b** Western blot showing the expression of BMAL1 in cellular compartments and whole cell lysates of mice expressing exclusively P1-HNF4α (WT) or P2-HNF4α (α7HMZ mice) (PonS = ponceau S). **c** Immunofluorescence of control tissue and metastasized human HCC specimens with antibodies specific to P1-HNF4α, P2-HNF4α, or to P1/P2-HNF4α. **d** Western blot showing P2-HNF4α and P1-HNF4α localization to the soluble nuclear compartment or the cytoplasm following overexpression of P2-HNF4α in AML12 cells. **e** FKPM counts from RNA-seq for *Src*, *Myc*, *Fgfr1*, *Mmp14*, and *Cdh1* mRNAs in WT or αHMZ livers. **f** FKPM counts from RNA-seq for *Ctnnbip1*, *Serpinf1*, *Axin1*, and *Porcn* in WT or αHMZ livers. ^*^P < 0.05, ^**^P < 0.01, ^***^P < 0.001, ^****^P < 0.0001, (*N* = 3). Scale bar is 50 µm. Error bars = SEM

Based on the specific circadian expression of the P2-HNF4α isoform and its inverse pattern of expression with BMAL1 in the context of HNF4α-expressing HCC, we examined the subcellular localization P1-HNF4α and P2-HNF4α in HCC under conditions in which they are co-expressed. P1-HNF4α, but not P2-HNF4α, has previously been demonstrated to be phosphorylated directly by SRC kinase^40^, which leads to trafficking from the nucleus to the cytoplasm. Subcellular fractionation of AML12 cells and HCC lines revealed that while nontransformed AML12 cells express P1-HNF4α only in the nuclear compartment, P1-HNF4α is present in the cytoplasmic fraction of cancer cells (Fig. 5a). In contrast, P2-HNF4α resides in the nuclear and chromatin compartments of HCC cells (Fig. 5a). Based on the inverse expression of P2-HNF4α and BMAL1 in liver cancer, liver samples were attained from P2-HNF4α-only expressing mice (α7HMZ mice)^50^ to determine whether BMAL1 levels were affected in an otherwise normal liver. Fractionation of livers from α7HMZ mice revealed that BMAL1 was reduced in both the cytoplasmic and nuclear compartments, and also in whole cell lysates (Fig. 5b; for quantification, see Supplementary Fig. 5A).

To further determine the degree to which P1-HNF4α subcellular localization is impacted in HCC, human HCC was stained with antibodies specific for either the P1- or P2-HNF4α isoforms and compared to sections stained with the P1/P2-HNF4α antibody. The staining revealed that while only P1-HNF4α is expressed in normal tissue, P2-HNF4α is predominantly expressed in HCC specimens (Fig. 5c and Supplementary Fig. 5b). While some P1-HNF4α was detected in human HCC, nuclear staining was considerably reduced compared to control tissue (Fig. 5c). To determine whether expression of P2-HNF4α can affect P1-HNF4α subcellular localization, AML12 cells were transfected with P2-HNF4α (HNF4α8) and analyzed by immunofluorescence (Supplementary Fig. 5c) and by Western blot analysis (Fig. 5d). P2-HNF4α overexpression resulted in a pronounced cytoplasmic export of P1-HNF4α, which was accentuated when cells were treated with MG132 (Fig. 5d and Supplementary Fig. 5c). Thus, under conditions of P1-HNF4α and P2-HNF4α co-expression, P2-HNF4α becomes the predominantly nuclear-localized isoform (Fig. 5d).

To determine whether livers expressing only P2-HNF4α showed global alterations in gene expression consistent with those observed in P2-HNF4α-expressing HCC, RNA-seq was performed on WT and α7HMZ livers at three different *zeitgeber* times. A comparison of the normalized FPKMs (Fragments Per Kilobase of transcript per Million mapped reads) revealed a pronounced upregulation of *Fgfr1*, a receptor known to be associated with HCC progression and metastasis^53^ (Fig. 5e). Similarly, the matrix metallopreoteinase *Mmp14*, whose transcriptional regulation by HNF4α has been reported to be involved in metastasis^54^, was increased at several time points in α7HMZ mice. α7HMZ livers showed a downregulation of *Cdh1* at some time points, suggesting an altered circadian phase in the absence of P1-HNF4α. Interestingly, expression of *Src* was elevated in α7HMZ livers, which could in part explain the nuclear export of P1-HNF4α in P2-HNF4α-positive HCC^40^. Finally, *Myc* abundance was increased in P2-HNF4α expressing livers, consistent with increased proliferation of HCC expressing the P2 isoform.

HNF4α has previously been linked to the Wnt/β-catenin pathway and the isoforms show distinct recruitment to genes involved in Wnt/β-catenin signaling^30^. To examine whether Wnt/β-catenin signaling might be different in α7HMZ livers, RNA-seq data was analyzed for changes in expression of genes involved in this pathway. Several Wnt/β-catenin pathway genes were significantly altered between WT and α7HMZ livers, with a pronounced upregulation of the positive regulator *Porcn* in α7HMZ livers, but a downregulation of several negative regulators of the pathway, including *Ctnnbip1*, *Serpinf1*, and *Axin1* (Fig. 5f). Interestingly, *Serpinf1* (also known as *Pedf*) has been shown to negatively regulate the Wnt/β-catenin pathway in the liver specifically^55^ and is reported to have antiangiogenic activity in the context of HCC^56^. Thus, downregulation of *Serpinf1* in the context of α7HMZ livers is consistent with P2-HNF4α activation of the Wnt/β-catenin pathway and promoting tumor growth.

### Direct and indirect repression of BMAL1 by P2-HNF4α

**Fig. 6:**
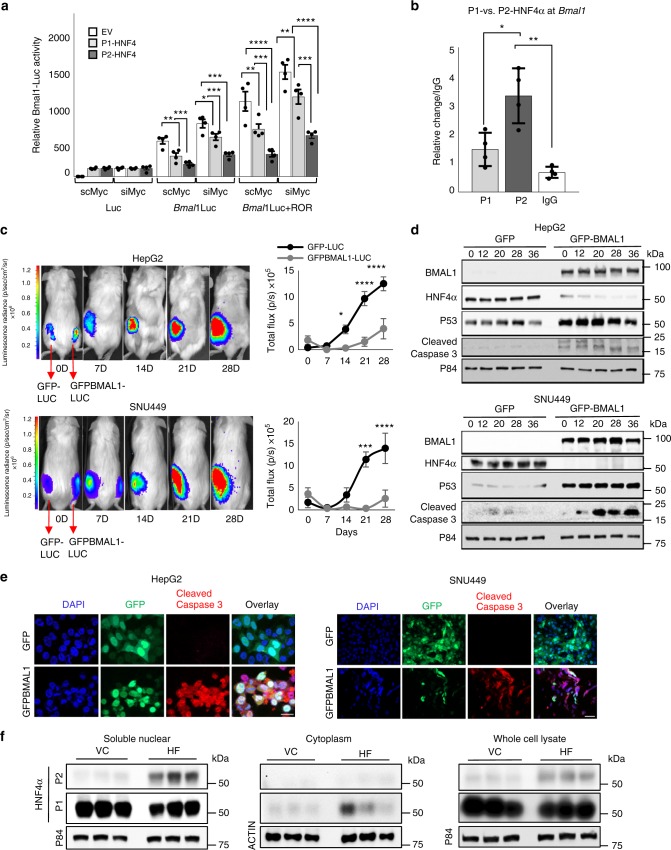
BMAL1 overexpression in HNF4α-positive HCC inhibits tumor growth **a** Luciferase assay results showing luciferase expression from BMAL1-LUC following transfection of DNA for the empty vector (EV), *P1-Hnf4a* or *P2-Hnf4α* with or without co-expression of RORα and with co-application of scrambled or *Myc* siRNA oligonucleotides. Comparing EV to P1/P2-HNF4α. Two-way ANOVA, Sidak’s multiple comparisons test, *P < 0.03, **P < 0.005, ***P < 0.0005, ****P < 0.0001, (*N* = 6). **b** Chromatin immunoprecipitation (ChIP) of P1- or P2-HNF4α in HepG2 cells followed by qPCR reveals amplification of *BMAL1* sequence upstream of the transcriptional start. **c** In vivo bioluminescence of hepatoblastoma and HCC tumors in immune compromised mice on days 0, 7, 14, 21, and 28 after subcutaneous injection of HepG2 or SNU449 cells expressing vectors *Luc* and *Gfp* or *Luc* and *Gfp-Bmal1*. Quantification of tumor size, right panel. Two-way ANOVA, Sidak’s multiple comparisons test, *P < 0.03, **P < 0.005, ***P < 0.0005, ****P < 0.0001. (*N* = 6–9). Scale bar is 100 µm. **d** Western blot reveals the abundance of BMAL1, P1/P2-HNF4α, P53, cleaved caspase 3, and P84 after serum synchronization of HepG2 and SNU449 cells previously transfected with *Gfp* or *Gfp-Bmal1*. **e** Staining of HepG2 and SNU449 cells 48 h after infection with virus containing *Gfp-Bmal1*, using antibodies to GFP and cleaved caspase 3. Overlay with DAPI nuclear stain. Scale bar is 20 µm. **f** Western blot showing P2-HNF4α and P1-HNF4α localization in the soluble nuclear and cytoplasmic cellular compartments, or in whole cell lysates of livers from animals with diet-induced obesity, using antibodies specific to P2-HNF4α or P1-HNF4α. Error bars = SEM

To elucidate what mechanisms might be responsible for the incompatibility of BMAL1 and HNF4α in HCC, we determined whether HNF4α regulates the transcription of the *BMAL1* promoter. Hepa-1c1c7 cells were transiently transfected with P1- or P2-HNF4α expression constructs and a *BMAL1-LUC* reporter with or without the BMAL1 activator, retinoic acid receptor related orphan receptor A (RORα). Because prior studies have revealed that MYC can repress BMAL1 expression in specific cancer cells^14,57^, we co-transfected the cells with si*Myc* or scrambled oligonucleotides to determine the extent to which HNF4α repression of *Bmal1*could be indirect through changes in *Myc* expression. While both P1- and P2-HNF4α reduced basal and RORα-activated BMAL1-driven LUC expression, P2-HNF4α had a much stronger repressive effect, reducing the RORα-induced activation to near baseline levels (Fig. 6a). Compared to scrambled oligonucleotides, the addition of si*Myc* contributed a further ~16 and 7% reduction over P1-HNF4α and P2-HNF4α repression of Bmal1-luc, respectively (Supplementary Fig. 6A). ChIP-seq reveals that P1-HNF4α binds the murine *Bmal1* promoter at approximately 350 base pairs upstream of the transcriptional start site (Supplementary Fig. 6b). To confirm whether the distinct isoforms of HNF4α interact with the *ARNTL* locus in normal and cancer cells, HNF4α ChIP was performed on HepG2 cell extracts (Fig. 6b and Supplementary Fig. 6d) and mouse liver (Supplementary Fig. 6c). Antibodies specific to each isoform revealed that P2-HNF4α shows superior binding to *ARNTL* in human HepG2 cells (Fig. 6b). ChIP of normal liver using the P1/P2-HNF4α antibody confirmed that P1-HNF4α can also bind to the *Arntl* promoter (Supplementary Fig. 6c). (For ChIP in HepG2 cells using the P1/P2-HNF4α antibody, see Supplementary Fig. 6d). Thus, HNF4α both binds the *BMAL1* promoter and represses *BMAL1* expression at the transcriptional level, with the P2 isoform providing the primary nuclear repression in HCC cells (Fig. 5b–e). Thus, we conclude that while *Myc* upregulation in response to loss of nuclear P1-HNF4α may contribute marginally to BMAL1 loss in HCC, the primary mechanism involves direct transcriptional repression by P2-HNF4α.

### Ectopic BMAL1 in HNF4α-positive HCC impairs tumor growth

To determine whether forced expression of BMAL1 in BMAL1-deficient, HNF4α-positive hepatoblastoma and HCC impairs tumor growth, AML12, HepG2, Huh7, and SNU449 cells were transfected with an expression vector for BMAL1 and analyzed for growth and viability. Ectopic expression of BMAL1 in HepG2 cells resulted in a transient loss of HNF4α expression (Supplementary Fig. 6h); and cancer cells, but not AML12 cells overexpressing BMAL1, showed reduced proliferative capacity over 48 h. (Supplementary Fig. 6e). Furthermore, overexpression of P1-HNF4α in Hepa-1c1c7 cells significantly impaired the ability of cells to form 3D spheroids after 10 days in Matrigel, as did the transient transfection of *GFP-Bmal1* in HepG2 cells (Supplementary Fig. 6f-g). To determine whether the growth of liver tumors in vivo could be impeded by ectopic BMAL1 expression, we injected immune compromised NSG (NOD.Cg-*Prkdc*^*scid*^ *Il2rg*^*tm1Wjl*^/SzJ) mice subcutaneously with HepG2 or SNU449 cells stably expressing luciferase and co-transfected with GFP-BMAL1 or empty vector. Bioluminescence was measured on day 0 to ensure similar cell numbers were injected, and tumor growth was monitored weekly for 4 weeks. Compared to cells expressing luciferase only, BMAL1-expressing HepG2 and SNU449 cells were significantly retarded in tumor growth, resulting in a complete lack of tumor growth or substantially smaller tumors (Fig. 6c and Supplementary Fig. 6i). Thus, we conclude that the co-expression of P2-HNF4α and BMAL1 in HCC is incompatible with tumor cell proliferation in vitro and in vivo.

To determine the mechanisms by which BMAL1 induces cell death in HNF4α-positive cancer cells, HepG2 and SNU449 cells were transfected with GFP or GFP-BMAL1. Expression of GFP-BMAL1 resulted in the induction of the tumor suppressor P53 as well as cleaved caspase 3 protein at all circadian time points tested following serum shock (Fig. 6d). Staining of unsynchronized cells transfected with GFP or GFP-BMAL1 revealed an increase in cleaved caspase 3 in BMAL1 overexpressing cells (Fig. 6e), suggesting that forced expression of the circadian protein BMAL1 in HNF4α-positive HCC inhibits tumor growth by inducing apoptosis, though additional BMAL1-mediated mechanisms may also contribute^57,58^.

## Discussion

**Fig. 7:**
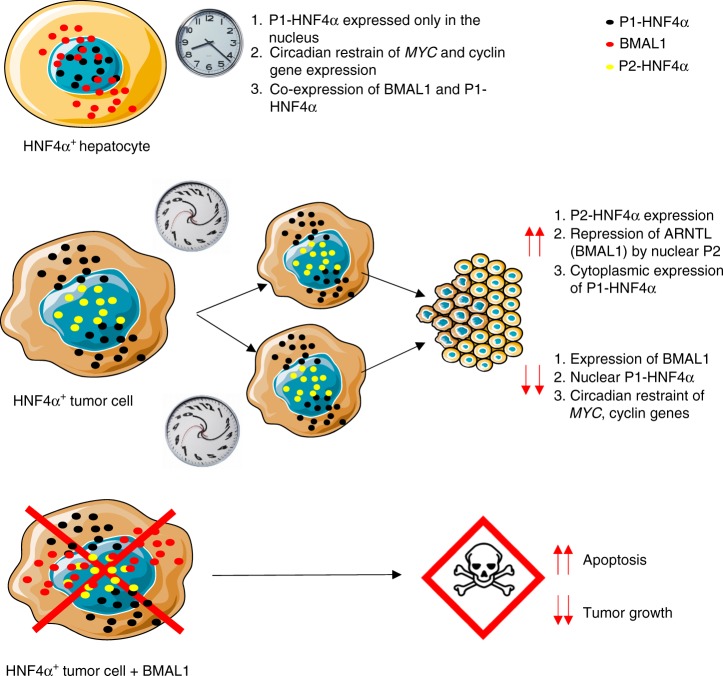
Model of HNF4α isoform and BMAL1 expression in normal vs. cancer cells. In normal hepatocytes, the P1-HNF4α isoform and the circadian protein BMAL1 are concomitantly expressed. While BMAL1 is found in both nuclear and cytoplasmic compartments, P1-HNF4α is found exclusively in the nucleus, where it both activates and represses target genes, sometimes in a circadian manner. In HCC, P2-HNF4α is induced, resulting in either the dual expression of both P1-HNF4α and P2-HNF4α or only P2-HNF4α in HNF4α-positive tumors (approximately 50% of HCC). Induction of P2-HNF4α results in direct transcriptional repression of BMAL1 and a large portion of P1-HNF4α becomes cytoplasmic, reducing its ability to repress target cyclin and EMT genes in a circadian manner. Forced expression of BMAL1 in HNF4α-positive HCC results in a cell death and inhibition of tumor growth

Our results reveal that the P1 and P2 isoforms of HNF4α have distinct circadian roles. In addition, they show that, as in colon cancer, P1-HNF4α is tumor suppressive, while P2-HNF4α is not. These data provide a model by which the upregulation of P2-HNF4α is causal for downregulation of BMAL1 expression in human HCC, consistent with findings in exon swap mice expressing P2-HNF4α in normal liver (Fig. 5). Furthermore, these data reveal that forced expression of BMAL1 inhibits HNF4α-positive tumor growth (Fig. 7). Taken together, these results suggest that targeting the circadian clock in HCC may be a promising treatment for the growth and progression of HCC tumors.

Several interesting scenarios regarding P2-HNF4α expression in HCC are plausible. Firstly, in spontaneous human HCC, P2-HNF4α is selectively induced^39^ by yet unidentified mechanisms. Interestingly, the proto-oncogene SRC can phosphorylate and down-regulate P1-HNF4α. Because P1-HNF4α represses the P2 promoter^30^, elevated SRC could potentially be leading to the induction of P2-HNF4α in HCC. SRC has been found to be overexpressed in HCC, and SRC inhibitors are a first-line chemotherapeutic treatment for liver cancer, although some patients are refractory to the treatment^59^. Our data suggest that P2-HNF4α increases SRC expression, which may provide a positive feedback loop in HCC.

Since P2-HNF4α expression results in an increase in cytoplasmic P1-HNF4α (as does SRC-induced phosphorylation), circadian repression in the nucleus of *MYC, CCND1*, *CCND1*, among other genes normally repressed in a healthy liver by P1-HNF4α appears to be reduced in HCC. However, ectopic expression of P1-HNF4α in HCC can still produce circadian repression of these targets, and knockdown of P1-HNF4α in HNF4α-positive HCC can increase the expression of these targets. In addition to targeting HCC by increasing BMAL1-mediated clock function, HCC growth could potentially be prevented by inhibiting P1-HNF4α from exiting the nucleus.

While HNF4α and BMAL1 are robustly co-expressed in normal hepatocytes, of significance is the distinct downregulation of BMAL1, which has been demonstrated to play tumor suppressive roles^14,57,58^ in HNF4α-positive HCC. The data presented here reveal that P2-HNF4α contributes to this by providing strong transcriptional repression of the *Bmal1* gene. While P1-HNF4α has a modest ability to repress *Bmal1* in luciferase assays in vitro, P2-HNF4α provides much stronger repression of *Bmal1* and furthermore, appears to be the primary nuclear-localized isoform in the context of HCC (see Fig. 5). Preferential binding of P2-HNF4α over P1-HNF4α to one or more corepressor proteins may contribute to greater repression of BMAL1. Consistent with what has been observed in the colon^28^, we find that the P1-HNF4α isoform preferentially binds the nuclear receptor ESRRA (Supplementary Fig. 7c). On the other hand, P2-HNF4α appears to preferentially bind PROX1 (Supplementary Fig. 7c), a protein known to interact with the HDAC3 repressive complex^60^. However, we have not yet confirmed whether these isoform-specific interactions contribute to differential repression of BMAL1. It is also conceivable that P2-HNF4α preferentially interacts with chromatin modifiers or with other transcriptional repressors that result in repression at specific target genes.

HCC appears to fall into two categories as it relates to HNF4α expression: either P2-HNF4α is induced so that the cells express both P1-HNF4α and P2-HNF4α or only the P2 isoform, or tumors are devoid of HNF4α expression altogether. At least for HNF4α-positive HCC, these data reveal the therapeutic potential of targeting the circadian clock in tumor progression. For example, there are known molecules that enhance clock function directly through the activation of a specific BMAL1 transcriptional regulator^61^, and thus it is worth determining whether such molecules might be employed as interventions in HNF4α-positive HCC. Furthermore, specific ligands have been proposed for HNF4α^62–64^. HNF4α has been shown to bind its endogenous ligand, linoleic acid, in a reversible fashion, indicating that HNF4α is a potential drug target^63^. Circadian targeting of normal vs. HCC cells with such ligands could provide a unique opportunity for tumor prevention or treatment if compounds that act in an isoform-specific fashion could be identified.

While HCC incidence in humans has historically been associated with viral infections, emerging evidence identifies nonalcoholic fatty liver disease and nonalcoholic steatohepatitis as major contributors to the increase in HCC^65,66^. Interestingly, we find an upregulation in the expression of the P2 isoform in mice chronically fed a high-fat diet (HFD) (Fig. 6f and Supplementary Fig. 7a), as well as a partial redistribution of the P1 isoform from the nucleus to the cytoplasm. Similarly, we find an induction of the P2 isoform in the livers of *db/db* mice, which are known to exhibit steatosis and fibrosis (Supplementary Fig. 7b). This suggests that aberrant expression of P2-HNF4α may be involved in the pathogenesis of HCC. Importantly, circadian disruption mimicking human jet-lag in mice induces spontaneous HCC via the development of fatty liver^17^, and we consider circadian disruption in humans to be an inadequately studied link to the increase in HCC, but of considerable translational value. Thus, circadian targeting of HCC holds great promise and may have advantages over traditional therapies due to decreased toxicity in hepatic and extra-hepatic tissues. Future studies investigating the clinical utility of circadian intervention in HCC are essential and highly promising.

## Methods

### Cell culture

Cell lines were obtained from the American Tissue Culture Collection (ATCC, Manassas, VA). SNU449, HepG2, Huh7, Hep3B, and Hepa-1c1c7 cells were grown in Eagle’s Minimal Essential Medium, and AML12 cells were grown in a 1:1 mixture of Dulbecco’s Modified Eagle’s Medium and Ham’s F12 medium. Media was supplemented with 0.005 mg ml^−^^1^ insulin, 0.005 mg ml^−1^ transferrin, 5 ng ml^−1^ selenium, and 40 ng ml^−1^ dexamethasone. HEK 293T cells were grown in Dulbecco’s modified Eagle’s medium, supplemented with 10% fetal bovine serum, 100 units ml^−1^ penicillin G sodium, and 100 μg ml^−1^ streptomycin.

For circadian synchronization by serum, cells were cultured in normal media until 90–100% confluency was reached. Cells were treated with media containing 50% horse serum for 2 h. At the end of the serum shock treatment, cells were washed with PBS and the medium was replaced with normal assay medium. Application of 50% horse serum is considered ZT0; therefore, ZT0 refers to the unsynchronized condition.

### Human HCC tissue arrays

Human tumor microarray slides containing lesions from patients with stages I–IV Hepatocellular carcinoma and matched nonmalignant tissues were obtained from UsBiomax.

### Mouse HCC tissue slides

Paraffin slides containing liver sections from chronically jet-lagged mice with spontaneous hepatocellular carcinoma were obtained from Loning Fu^17^.

### Mice

Care of animals was in strict accordance with guidelines from the McGovern Medical School, UTHealth Institutional Animal Care and Use Committee. Mice were group housed in standard pathogen-free conditions and fed ad libitum with a standard mouse chow (PicoLab Rodent Diet 5053) and water. Animals were maintained in steady 12-h light/12-h dark cycles (24-h LD cycles). For experiments using livers from diet-induced obese mice, animals were fed with a diet containing 60% kcal from fat, Research Diets D12492, for 30 weeks starting at 8 weeks of age.

*Hnf4a*^*F/F*^ and *SA*^*+/Cre-ERT2*^ mouse lines were originally provided by Gonzalez^25^. To generate the conditional *Hnf4a*^*F/F*;*AlbERT2cre*^ mice, *Hnf4a*^*F/F*^ mice were crossed with the tamoxifen-inducible hepatocyte-specific *Cre* recombinase expressing mouse *SA*^*+/Cre-ERT2*^. Livers from 10-week-old male and female mice of each genotype were used for chromatin immunprecipitation.

Eight-week-old male NSG mice, NOD.Cg-Prkdcscid Il2rgtm1Wjl/SzJ were used for subcutaneous injections of HepG2 and SNU449 cells.

### Mouse liver samples

Liver samples from α7HMZ mice used in the study were provided by Frances Sladek. α7HMZ mice are described in (Briançon and Weiss, 2006). Three young adult males on standard lab chow, aged 16–20 weeks, were sacrificed at 10:30 (ZT 3.5), 13:30 (ZT 6.5) and 20:30 (ZT 13.5) (lights on at 7:00 and off at 19:00) for liver RNA-seq analysis.

Livers from C57BL/6J mice (The Jackson Laboratory, 000664) under normal chow (PicoLab Rodent Diet 5053) or made obese with HFD (Research Diets D12492) were obtained from mice at 10 months of age. Mice had been maintained on their respective diets from 2 months of age.

### Plasmid constructs

The sources for all plasmid constructs are presented in Supplementary Table 2. *Bmal1*, *Hnf4α2*, and *Hnf4α8* were subcloned into pLenti CMV GFP Puro and pEGFP-C1. pLenti CMV GFP Puro was digested using BamHI and SalI. pEGFP-C1 was digested using XbaI and BamHI or Sal1 and XbaI followed by insertion of full length *Hnf4α2, Hnf4α8*,or *Bmal1* amplified from their respective pcDNA vectors using primers designed with cohesive ends (Supplementary Table 9). Dephosphorylation of the linearized vector was performed using CIP (Alkaline Phosphatase, Calf Intestinal) followed by ligation of the vector with the corresponding fragment using a Quick Ligation kit for 15 min at room temperature according to manufacturer’s protocol.

### Transient transfection and siRNA,

Transient transfection of plasmid vectors (Supplementary Table 2) was performed with Lipofectamine 2000 according to the manufacturer’s protocol. Briefly, 1 × 10^6^ cells were transfected with 1 µg of plasmid using 5 µl of Lipofectamine 2000. Knockdown of human and mouse P1/P2-HNF4α, P1-HNF4α and P2-HNF4α was performed using siRNA duplexes^28^ purchased from Eurofins Genomics (Supplementary Table 3). Approximately 1 × 10^6^ cells were transfected with 25 pmol of siRNA and 5 μl of Lipofectamine RNAiMAX Transfection reagent. Scrambled siRNA with similar GC content was used as a control. Transfections were performed following the manufacturer’s protocol.

### Lentivirus production and titration, and generation of stable cell lines

High titer lentiviral vector stock was produced in HEK 293T cells by Lipofectamine 2000 Transient transfection. Briefly, 10 µg of transfer vectors, 5 µg of each packaging vector, and 2.5 µg of envelope vector (Supplementary Table 2) along with 50 µl of Lipofectamine were used to transfect 1 × 10^7^ HEK 293T cells. Supernatant harvests were performed every 16 h for a total of three harvests and then pooled. Supernatant was filtered through 0.45 µm pore size filters and concentrated using one volume of Lenti-X concentrator reagent in three volumes of clarified supernatant according to the manufacturer’s protocol. The titration of lentivirus was assessed using a qPCR based approach. Woodchuck Hepatitis Virus Posttranscriptional Regulatory Element and albumin genes were used as targets for a SYBR green-based real-time qPCR method to determine virus titer^67^ (Supplementary Table 4). For stable cell line production, cells were infected with concentrated lentiviral stock at ~50 multiplicity of infection in the presence of 8 mg ml^−1^ polybrene for 8 h at 37 °C. A 2 µg ml^−1^puromycin was then added to eliminate uninfected cells. Media was replaced every 3–4 days with fresh, puromycin-containing medium. Stable lines were used as 3D spheroid cultures and injected into the xenograft model.

### Transwell invasion assays

Cells were seeded into the upper chamber of a Transwell insert in serum-free medium at a density of 50,000 cells/well. For invasion assays, inserts were precoated with 2 mg ml^−1^ Matrigel. Medium containing 20% FBS was placed in the lower chamber as a chemoattractant, and cells were incubated for 24 h in a CO_2_ incubator. Non-migrating cells were removed from the upper chamber by scraping. Remaining cells were fixed with methanol for 10-min and stained with 0.1% crystal violet. Cells were imaged using a Zeiss inverted light microscope and 4 randomly chosen fields chosen on the lower side of the filter were used for counting.

### 3D spheroid culture

For 3D Matrigel-embedded cultures, human and mouse HCC cells were trypsinized and resuspended in complete medium. Twenty-five microliter cell suspension containing 5 × 10^3^ cells was added to 25 μl of Matrigel with a protein concentration ranging from 7 to 7.5 μg μl^−1^. Matrigel/cell suspension was gently mixed and deposited into 24-well uncoated plates (Nalge Nunc International). To grow single organoids via the hanging drop method^68^, the lid from a 60 mm tissue culture plate was removed and 5 ml of PBS was placed in the bottom of the dish to act as a hydration chamber. The lid was inverted and 10 μl media containing 1 × 10^3^ cells was deposited onto the bottom of the lid. The lid was returned to its upright position on top of the PBS-filled bottom chamber and incubated at 37 °C overnight. This cell drop was carefully removed via pipet and mixed with 50% Matrigel prior to being deposited on the bottom of an uncoated 24-well plate. In both conditions, deposited Matrigel drops were incubated at 37 °C for 30 min. until solidified and then covered with 1 ml complete medium and grown at 37 °C under 5% CO_2_ for 15 days. The media was changed every 2 days^69^. Organoids were harvested and washed 4× in ice cold PBS to remove all Matrigel after a 15 day growth period, and subjected to protein analysis.

### MTT assay

Cell viability was assessed by MTT cell proliferation assay using a CellTiter 96^®^ Non-Radioactive Cell Proliferation Assay (MTT) kit. Plates were processed according to the manufacturer’s instructions. Absorbance was read at 570 nm using a Tecan infinite M1000 reader.

### Luciferase reporter assay

To analyze *Bmal1* promoter activity, 1 μg of each construct was co-transfected into 1 × 10^6^ Hepa1-c1c7 cells using 5 μl of Lipofectamine 2000 according to the manufacturer’s manual. Luminescence was measured 48 h posttransfection using a Luciferase assay system. Briefly, cells were washed in PBS and lysed by rapid shaking on a plate shaker at 200 RPM for 10 min. at RT in Luciferase Lysis buffer (Supplementary Table 5). Thirty microliters of cell lysate was added to 70 μl of Luciferase React Buffer containing luciferin (Supplementary Table 5), and luciferase activity was measured immediately on a plate reader. The pGL3-Basic plasmid (promoter-less) was used in each experiment to determine the basal levels of luciferase expression. Each construct was tested in three independent transfection experiments. A *beta-galactosidase* measurement was used to normalize experiments for transfection efficiency using Lac Z. Beta-galactosidase activity was measured by adding 30 μl lysate to 70 μl of prepared Z buffer (Supplementary Table 5) followed by a 37 °C incubation for 5-min. Reactions were stopped with 50 μl of 1 M NaHCO_3_. Absorbance was read at 450 nm using a Tecan infinite M1000 plate reader.

### RNA extraction, reverse transcription, and quantitative PCR

Total RNA was isolated from cells and liver using TRIzol reagent according to the manufacturer’s protocol. One microgram of total RNA was used for cDNA synthesis using an iScript cDNA synthesis kit. Advanced Universal SYBR Green Super mix from Bio-Rad was used for qPCR amplification using a Bio-Rad C1000. PCR protocol settings were as follows: 95 °C for 30 s, 95 °C for 10 s, 62 °C for 30 s, and then 39 cycles at 65 °C for 31 s and 65 °C for 5 s. Ribosomal subunit 18 s expression was used as control. The fold change in mRNA expression for each gene was calculated using 2^−ΔΔC^. Primers used for amplification are presented in Supplementary Table 6.

### Fractionation and immunoblotting

For whole cell lysates, liver tissue was homogenized in RIPA lysis buffer (Supplementary Table 7) for 15 s, using a MagNA Lyser (Roche, IN, USA). Cultured cells were sonicated (Qsonica, Newton, USA) for 10 s at 30% amplitude in RIPA lysis buffer. Samples were spun at 10,000×*g* for 10 min to eliminate insoluble material. The total protein levels of the lysates were determined using the bicinchoninic acid method. Protein extracts were analyzed using 8% sodium dodecyl sulfate polyacrylamide gel electrophoresis followed by transfer to the nitrocellulose membrane before staining with primary antibodies (Supplementary Table 8). Secondary antibodies conjugated to horseradish peroxidase and enhanced chemiluminescence substrate, or alternatively, florescent conjugated secondary antibodies were used for detection (Supplementary Table 8). All full length blots and gels are supplied in the supplementary figures.

For tissue fractionations, liver tissue was homogenized for 15 s in BA (Supplementary Table 7) using a MagNA Lyser. Samples were spun at low speed for 10 min and supernatant was saved for cytoplasmic fractions. Pellets were resuspended in 1 ml of BA and spun at low speed for 20 min. Pellets were washed 2× with low-salt buffer (LSB) (Supplementary Table 7) and the final pellet was dissolved in 150 µl LSB per 100 µl pellet. The resulting homogenate was resuspended vigorously in 2× volume high-salt buffer (Supplementary Table 7), and nutated for 1 h at 4 °C. Samples were spun for 20 min at 10,000×*g* and the soluble nuclear material (supernatant) was snap frozen in liquid nitrogen and stored at −80 °C. Remaining pellets were resuspended in RIPA lysis buffer and sonicated (Qsonica, Newton, USA) for 10 s at 30% amplitude. Samples were spun at 10,000×*g* for 5 min to remove insoluble material and the resulting chromatin material was stored at −80 °C.

### Immunofluorescence

Cells and 3D spheroids were plated on glass cover slips in a six-well plate and fixed with 3.7% of methanol-free formaldehyde for 10 min. followed by permeabilization with 0.1% Triton X-100 for 5 min. Cells were blocked with 3% BSA dissolved in 1× PBS (pH 7.4) for 1 h. Cells were incubated overnight at 4 °C with specific primary antibodies (Supplementary Table 8) in 3% BSA. Human HCC microarray and mouse liver paraffin slides were baked for 15 min at 65 °C and samples were immersed in xylene for 20 min and sequentially rehydrated in 100, 90, 80, and 70% alcohol series and rinsed with PBS. For antigen retrieval, slides were steamed for 30 min in 10 mM citrate buffer (pH 6). After blocking with 10% goat serum and 3% BSA in PBS for 1 h, slides were incubated with primary antibody overnight at 4 °C (Supplementary Table 8). For both IF and IHC, primary staining was followed by appropriate secondary fluorescent antibody (Supplementary Table 8) at 1:500 in a dark chamber for 1 h at room temperature. Nuclei were stained using DAPI. Images were captured and processed using a Zeiss Fluorescent microscope and Zen software.

### CHIP assay

Chromatin immunoprecipitation was performed as described in ref. ^70^ with some modifications. One hundred milligrams of frozen tissue or cells in culture from a 15 cm dish were washed in PBS containing protease inhibitors (1% NP-40, 1 mm PMSF, 1 mM NaF, 400 mM NAM, and 3.3 mM TSA). Tissue was minced in PBS with inhibitors followed by cross-linking with DSG (2 mM final) in PBS containing MgCl (10 mM final) for 45 min at 25 °C. Formaldehyde (1% final) was added for additional cross-linking at 25 °C for 15 min. Samples were spun at low speed and resuspended in PBS with protease inhibitors for homogenization. Following homogenization, samples were spun at low speed and resuspended in 2 ml ChIP sonication buffer (1% Triton X-100, 50 mM Tris, pH 8.1, 5 mM EDTA, 0.1% deoxycholate, 150 mM NaCl) containing protease inhibitors. Following sonication, samples were spun at 16,000×*g* in a microcentrifuge and supernatants were diluted 1:4 in ChIP dilution buffer (0.01% SDS, 1.1% Triton X-100, 1.2 mM EDTA, 16.7 mM Tris [pH 8.1], 167 mM NaCl), and immunoprecipitated with HNF4α antibodies (Supplementary Table 4) or control IgG overnight. Protein:DNA complexes were captured with blocked protein G-agarose beads and following elution, cross-linking was reversed by heating at 65 °C overnight. Proteins were digested by 0.17 μg μl^−1^ proteinase K (New England Biolabs), and the DNA was extracted with phenol-chloroform, precipitated with ethanol, and dissolved in 100 μl Tris-EDTA buffer (10 mm Tris-Cl [pH 8.0], 1 mm EDTA). ChIP samples were diluted in 120 μl ddH_2_0. Six microliters of ChIP samples were used for qPCR analysis using gene specific primers (Supplementary Table 9).

### Xenograft experiments

HCC (SNU449) and hepatoblastoma cells (HepG2) stably expressing LUC were infected with pLenti-*Gfp* or pLenti-*Gfp-Bmal1*.  Forty-eight hours following infection, 2 × 10^6^ cells were implanted subcutaneously into the right and left flanks.

Animals were monitored for luciferase expression 6 h following injection and then weekly for four consecutive weeks. For bioluminescent monitoring, 100 μl of 50 mg ml^−1^ D-luciferin was injected intraperitoneally to the animals 15 min prior to luciferase measurements. Tumor growth was tracked by monitoring luciferase activity using an IVIS Spectrum (PerkinElmer, USA). Resulting tumors were excised at 4 weeks.

### Tamoxifen injections

*Hnf4a*^*F/F*; *AlbERT2cre*^ mice were injected intraperitoneally with tamoxifen (10 mg ml^−1^) in corn oil for 5 consecutive days (days 1–5) and then allowed to rest for 10 days prior to euthanization at the indicated zeitgeber times.

### Quantification and statistical analysis

All results are expressed as the mean ± SEM. Experiments with one variable were analyzed by unpaired Students *t* tests or one-way ANOVA using Dunnetts multiple comparison test. Experiments involving two variables were analyzed by two-way ANOVA using Bonferroni post-tests (Prism 7.0). Significance was defined as a *P* < 0.05. To test for rhythmicity, JTK_Cycle^48^ was applied, using a window of 20–28 h to capture circadian oscillations. For serum shock experiments, rhythmicity was ascertained from all values excluding the ZT0 (unsynchronized) time point.

### Fluorescent quantification

Fluorescent intensity of HNF4α or BMAL1 was measured using Fiji (Image J). Briefly, areas of interest were selected using the Drawing tools. From selected cells, the mean fluorescent densities were measured using “set measurement” in the analysis menu. Values were correcting using the total cell fluorescence (CTCF) values. CTCF = mean fluorescent density of cells − mean fluorescent density of background readings.

### Expression profiling (RNA-seq) and analysis

RNA sequencing (RNA-seq) was carried out as previously described^30^. WT and α7HMZ male mice were sacrificed (*N* = 3, aged 16–18 weeks), at one of three time points, 10:30 am, 1:30 pm, 8:30 pm (ZT 3.5, ZT 6.5, and ZT 13.5, respectively). The three mice were harvested in succession within a 30 min time frame. Livers were weighed and rinsed in PBS. Two ~25 mg pieces from each liver were immediately frozen in liquid nitrogen and stored at −4 °C. The miRNeasy Mini Kit (Qiagen) was used to extract and purify total RNA; 4 µg of each sample was used to prepare a poly(A) + RNA library using TruSeq RNA Sample Prep v2 Kit (Illumina, Cat# RS-122-2001). Libraries submitted for 75 bp single-end sequencing with Illumina NextSeq 500 at the UCR IIGB Genomics Core. A total of 24 libraries (3 fed time points, 1 fasted time point, 2 genotypes each, and 3 replicates) were multiplexed and sequenced in two separate runs each of which yielded ~600 M reads, averaging ~50 M reads per sample.

Reads were aligned to the mouse reference genome, mm10, with Illumina’s iGenome genes.gtf file using TopHat v2.1.1 with default parameters with the exception of allowing only one unique alignment for a given read, instead of the default 20. Raw read counts were calculated at the gene level for each sample using HTSeq v0.6.1. Library normalization was performed with EDASeq. Within-lane normalization on GC content was performed with the loess method and between-lane normalization was performed with the nonlinear full quantile method. Normalization factors from EDASeq were used for differential expression analysis with DESeq2. Normalized read counts, FPKM (fragments per kilobase per million), and rlog (regularized log transformation) results were generated for downstream analysis. Pairwise contrasts for differential gene expression were generated for all relevant comparisons. Sample distance matrix were generated using rlog transformed values from DESeq2.

### Detailed reagent information

Specific details related to all reagents and antibodies used can be found in Supplementary Table 10.

### Image creation

Underlying cell images in Fig. 7 were taken from the Library of Science and Medical Illustraions, at http://www.somersault1824.com/resources/. A link to the license address is: https://creativecommons.org/licenses/by-nc-sa/4.0/.

## Electronic supplementary material

Supplementary Information

## Data Availability

All data are publicly available. The genome-wide RNA-seq data has been deposited into Gene Expression Omnibus (GEO) and has a number of GSE117972. All relevant data are available from the authors associated with this manuscript and will be disseminated upon request.
