# Supplementary material for: Incompatibility of the circadian protein BMAL1 and HNF4α in hepatocellular carcinoma
Source: Nat Commun. 2018 Oct 19;9:4349. doi: 10.1038/s41467-018-06648-6 (PMC6195513; doi:10.1038/s41467-018-06648-6)
Supplement: Supplementary file 1 — Supplementary Information [file 41467_2018_6648_MOESM1_ESM.docx]

**Supplementary Information for “Incompatibility of the Circadian Protein BMAL1 and HNF4α in Hepatocellular Carcinoma” Fekry et al.**

**
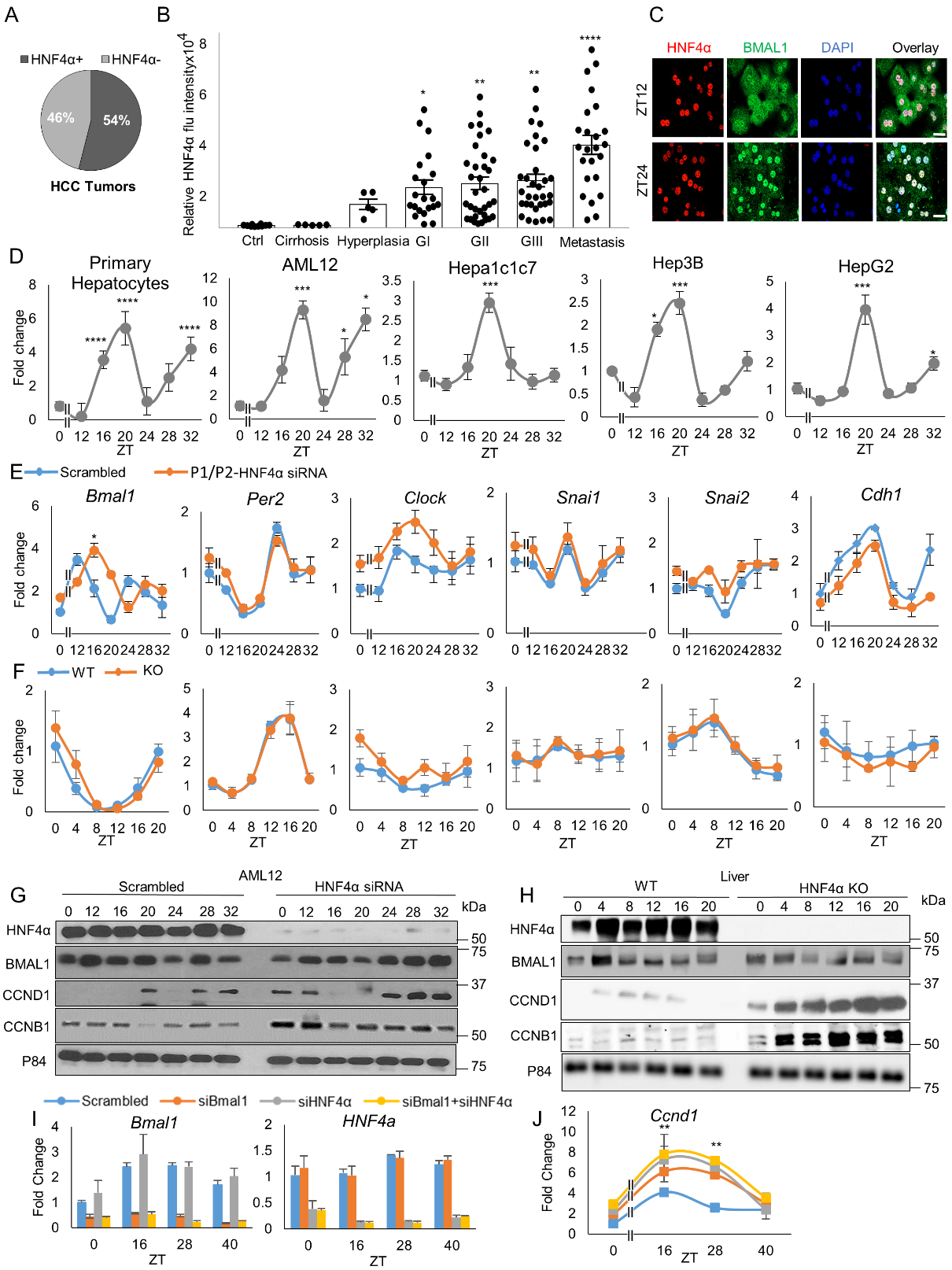
**

**Supplementary Figure 1 Loss of HNF4α Alters Circadian Expression of HNF4α Target Genes.**  (A) Percent of human HCC tumors that express P1/P2-HNF4α or are devoid of expression. (B) Relative intensity of P1/P2-HNF4α protein across human HNF4α-positive tumors of varying grades as well as metastasized lesions. Compared to controls at the same time: *P<0.03, **P<0.005, ***P<0.001, ****P<0.0001, *One-way ANOVA* test, Dunnett’s multiple comparisons test. (N=8-43. (C) Staining of fixed primary hepatocytes harvested 12-h or 24-h following serum shock, using antibodies to BMAL1 and P1/P2-HNF4α. (Scale bar 50µm). (D) RT-PCR reveals expression of the CLOCK: BMAL1 target gene, *Dbp*, in primary hepatocytes, non-transformed AML12 cells, and hepatoblastoma and HCC lines Hepa-1c1c7, Hep3B, and HepG2, *in vitro* after circadian synchronization by serum shock. Compared to ZT0 at the same time: *P<0.01, **P<0.01, ***P<0.001, ****P<0.0001, *One-way ANOVA* test, Dunnett’s multiple comparisons test. (N=4). (E) RT-PCR of serum-shocked AML12 cells transfected with scrambled or siRNA oligonucleotides for P1/P2-HNF4α isoforms reveals expression of core clock genes *Bmal1*, *Per2*, *Clock, Snai1,Snai2* and *Cdh1* at different *zeitgeber* times following serum shock. (F) RT-PCR reveals expression of *Bmal1*, *Per2*, *Clock, Snai1, Snai2* and *Cdh1* in livers of WT and liver-inducible HNF4α knockout (KO) animals throughout the circadian cycle. (G-H) Western blot showing P1/P2-HNF4α, BMAL1, CCND1, CCNB1and P84 expression in Aml12 cells and in WT and HNFα KO liver. (I-J) RT-PCR reveals the expression of *Bmal1, Hnf4α* and *Ccnb1* following serum shock and prior knockdown of P1/P2-HNF4α and BMAL1 singly or together with specific siRNA. *Two-way ANOVA*, Sidak’s multiple comparisons test, *P<0.01, **P<0.005,***P<0.001, ****P<0.0001. (N=4). (For JTK_Cycle rhythmicity statistics, see Table S1.) Error bars=SEM.

*
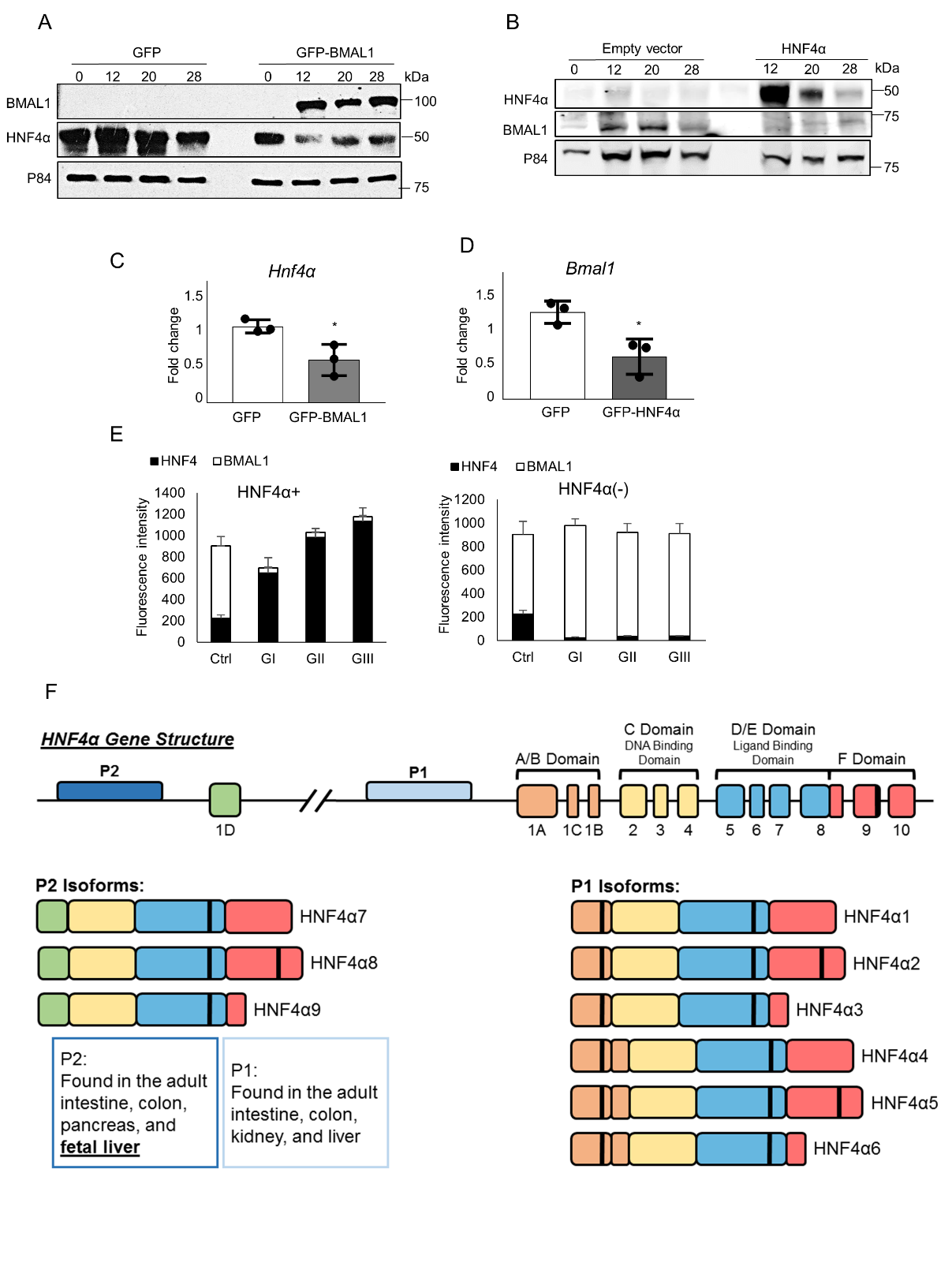
*

**Supplementary Figure 2 Incompatibility of BMAL1 and HNF4α in HCC but not Healthy Liver.** (A) Western blot showing P1/P2-HNF4α and BMAL1 protein in BMAL1-negative HepG2 spheroids following overexpression of BMAL1 using lentiviral infection. (B) BMAL1 and HNF4α expression in HNF4α-negative Hepa-1c1c7 3D spheroids after infection with expression vector for P1/P2*-Hnf4a*. (C) RT-PCR reveals amplification of P1/P2*-Hnf4a* in sorted GFP-positive HepG2 cells (left panel) transfected with *Gfp* empty vector (“Gfp”) or *Gfp-Bmal1*. (D) RT-PCR reveals relative *Bmal1* abundance in sorted GFP-positive cells following transfection of Hepa-1c1c7 cells with *Gfp* or *Gfp-P1/P2-Hnf4a*. Unpaired Two tailed Student T-Test,*P<0.05. (E) Quantification of BMAL1 and P1/P2-HNF4α in control tissue and in human HCC (grades I-III) that are P1/P2-HNF4α-positive or P1/P2-HNF4α-negative (N=8-15 per group). (F) Schematic of the HNF4α gene structure.

**
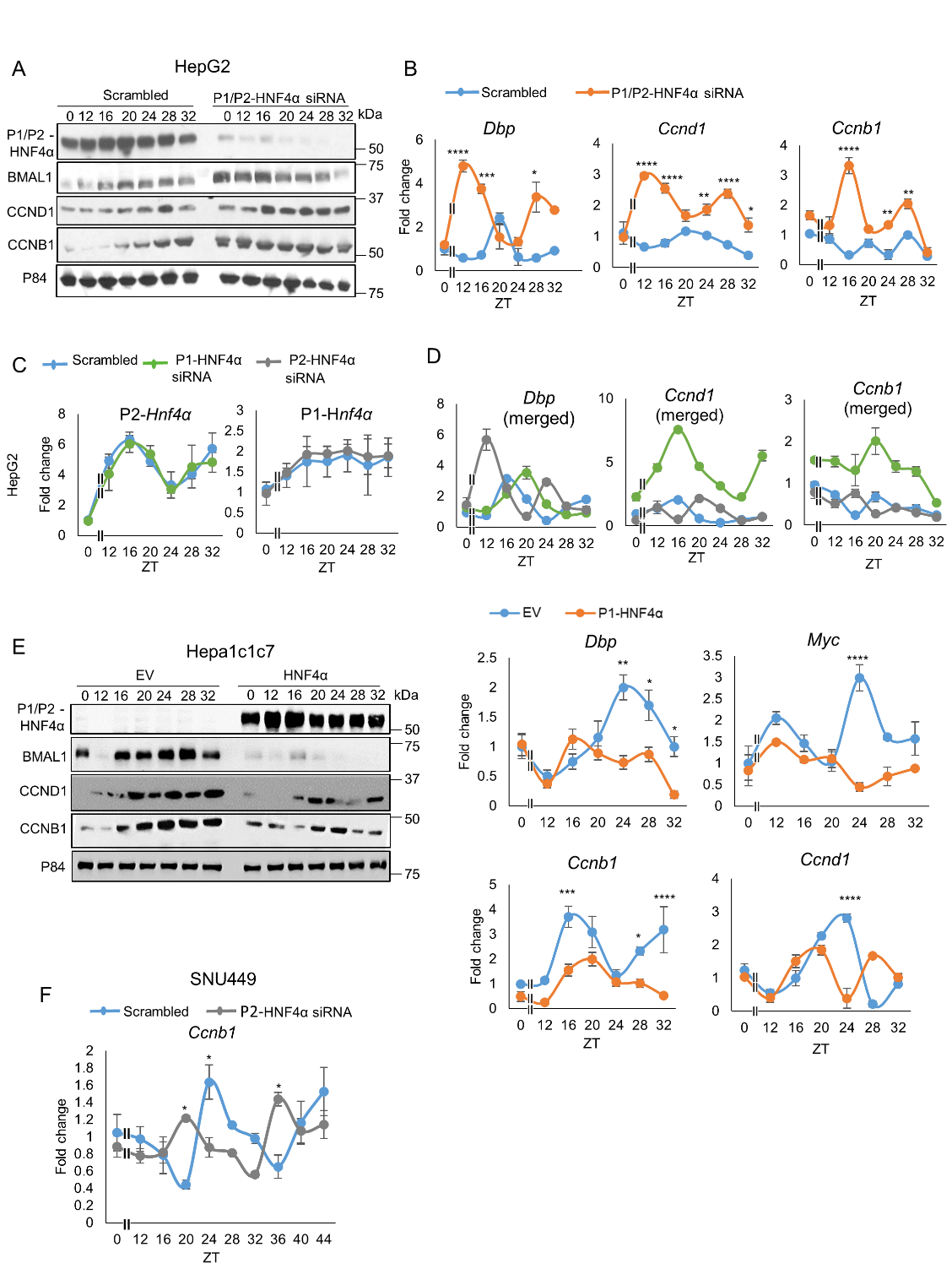
**

**Supplementary Figure 3 Isoform-Specific Circadian Effects of HNF4α on Cyclin Gene Expression** (A) Western blot showing P1/P2-HNF4α, BMAL1, CCND1, and CCNB1 expression in HepG2 cells serum shocked and previously treated with siRNA specific to both P1/P2-*Hnf4a* isoforms or with scrambled oligonucleotides. (B) RT-PCR reveals the expression of *Dbp*, *Ccnd1* and *Ccnb1* following serum shock and with or without specific knockdown of P1/P2-*Hnf4a* with specific siRNA. (C) RT-PCR reveals the expression of P1 or P2-*Hnf4a* following serum shock and knockdown of P2 or P1-*Hnf4a* with specific siRNA. (D) Merged gene expression for HepG2 cells treated with scrambled oligonucleotides or siRNA specific to the P1- HNF4α or P2- HNF4α followed by serum synchronization (E) Western blot showing P1-HNF4α, BMAL1, CCND1, and CCNB1 proteins at different time points following serum shock of *P1-Hnf4a*-transfected Hepa-1c1c7 cells (left panel). RT-PCR analysis reveals fold change in the mRNA abundance of *Dbp*, *Myc*, *Ccnb1* and *Ccnd1* following serum shock of previously *P1/P2-Hnfa*-transfected Hepa-1c1c7 cells (right panel). (F) RT-PCR reveals the expression of *Ccnb1*following serum shock in SNU449 cells with prior knockdown of *P2-Hnf4a* with specific siRNA or administration of scrambled oligonucleotides. *Two-way ANOVA*, Sidak’s multiple comparisons test, *P<0.03, **P<0.005, ***P<0.0005, ****P<0.0001. (N=4). (For JTK_Cycle rhythmicity statistics, see Table S1.) Error bars=SEM.

**
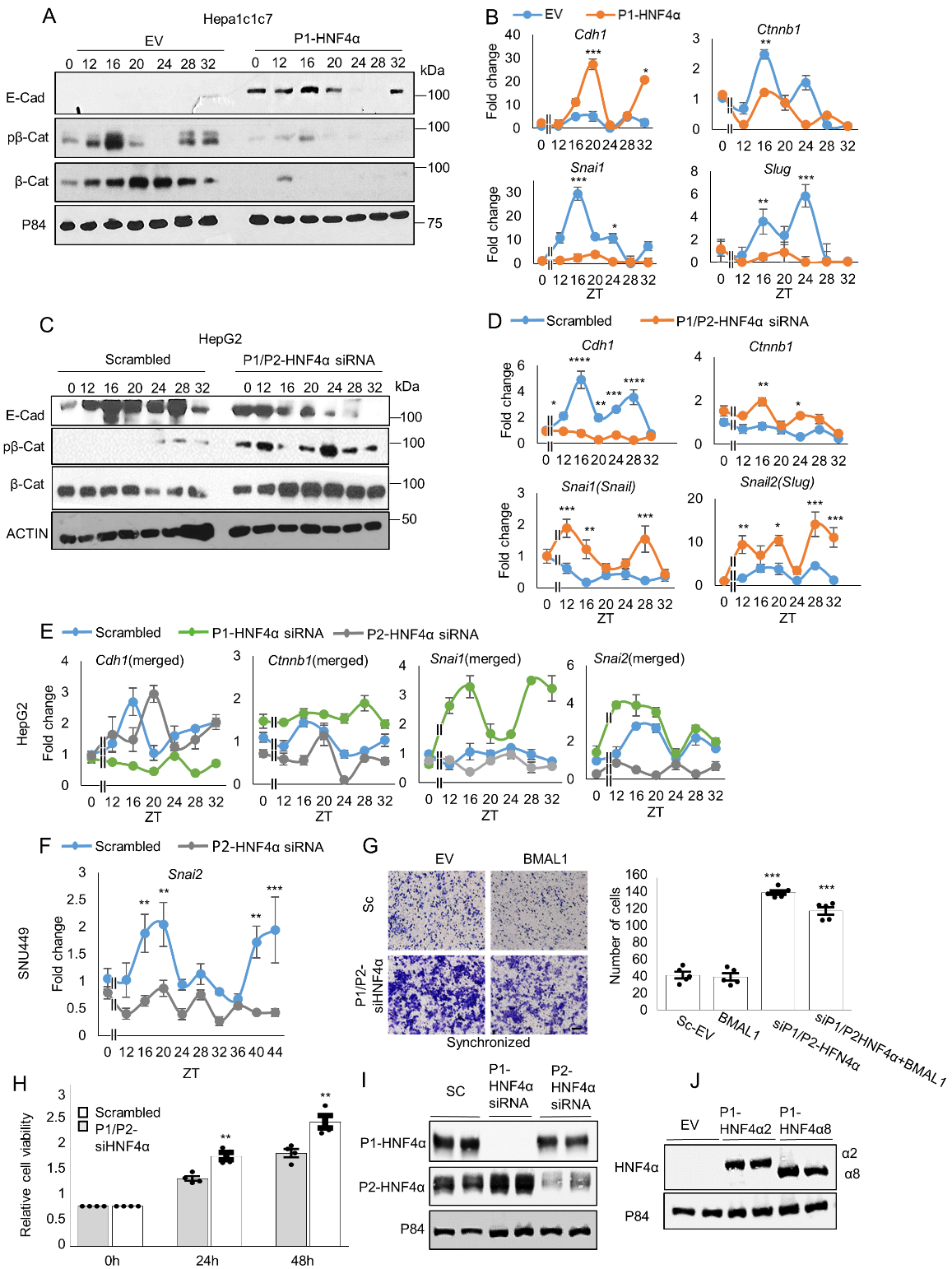
**

**Supplementary Figure 4 Forced Co-Expression of HNF4α and BMAL1 in HCC Inhibits Cell Proliferation and Invasion** (A) Western blot showing the post-serum shock expression of E-cadherin (CDH1), phospho-β-catenin and total β-catenin (CTNNB1) proteins in Hepa-1c1c7 cells transfected with empty vector (ev) or a P1*-Hnf4a* expression vector. (B) RT-PCR results reveal changes in expression over time following serum shock in the mRNA abundance of *Cdh1*, *Ctnnb1*, *Snail* (*Snai1)* and *Slug* (*Snai2)* following transfection of empty vector (ev) or the P1*-Hnf4a* isoform in Hepa-1c1c7 cells. Two-way ANOVA, Sidak’s multiple comparisons test, *P<0.03, **P<0.005, ***P<0.0005, ****P<0.0001, (N=4). (C) Western blot showing expression of CDH1, phosphorylated and total β-catenin (CTNNB1), after P1/P2- HNF4α knockdown followed by serum shock. (D) RT-PCR reveals mRNA abundance of EMT genes *Cdh1,* *Ctnnb1,* *Snai2* and *Snai1* after serum shock and prior application of scrambled oligonucleotides or siRNA recognizing P1/P2-HNF4α in HepG2 cells. (E) Merged RT-PCR data showing EMT gene mRNA abundance in cells treated with scrambled oligonucleotides (Sc), or siRNA to P1-HNF4α, or P2-HNF4α followed by serum shock. (F) RT-PCR reveals circadian expression of Snai2 following serum shock and prior application of scrambled or siRNA specific to P2-HNF4α. (G) Invasion assays shows invaded HepG2 cells transfected with empty vector (EV) or *Bmal1* expression construct in the presence or absence of siRNA targeting P1/P2-HNF4α. Quantification of invaded cells, right panel. (H) Cell proliferation (measured by MTT assay) of HepG2 cells transfected with scrambled oligonucleotides or P1/P2*-siHn4a*. (I) Western blot analysis of P1/P2-HNF4α after specific knockdown of P1- HNF4α or P2-HNF4α in HepG2 cells. (J) P1- and P2-HNF4α expression in Hepa-1c1c7 cells transfected with P1 or P2-specific *Hn4a* expression vectors. Quantification of invaded cells, right panel. Compare to SC or EV at the same time *P<0.05, **P<0.01, ***P<0.001, ****P<0.0001, *One-way ANOVA* test, Dunnett’s multiple comparisons test, (N=5), (Scale bar 100µm). (For JTK_Cycle rhythmicity statistics, see Table S1.) Error bar=SEM.


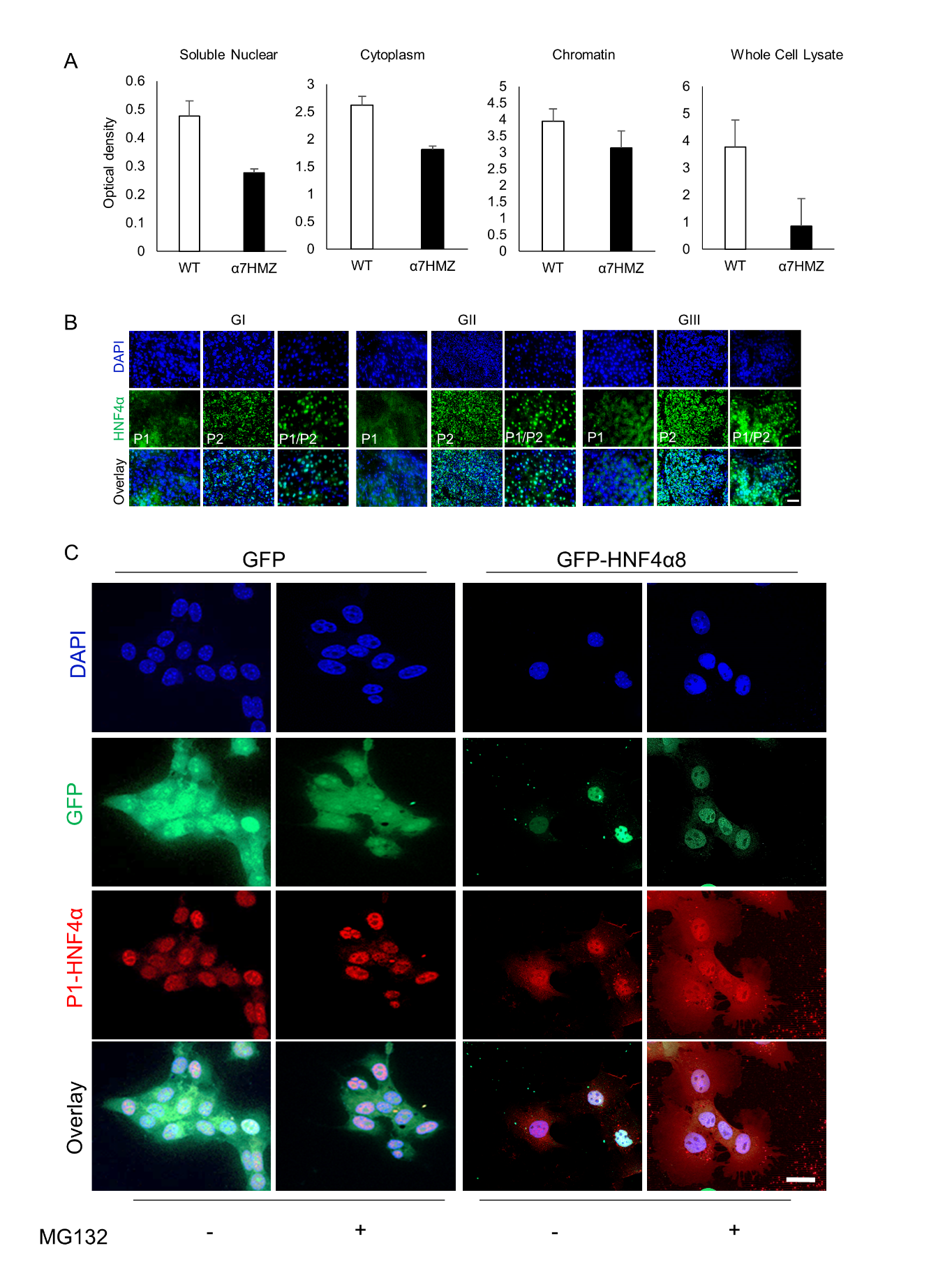


**Supplementary Figure 5 Expression of P2-HNF4α Results in the Cytoplasmic Accumulation of P1-HNF4α.** (A) Quantification of BMAL1 protein from western blots of WT and α7HMZ livers. (Corresponds to Figure 5B.) (B) Immunohistochemistry of grades 1-3 (GI, GII, GIII) human HCC specimens using antibodies specific only to P1-HNF4α, P2-HNF4α, or to P1/P2-HNF4α. (C) Immunocytochemistry of AML12 cells transfected with *Gfp*-empty vector of *Gfp-Hnf4α8* (*P2-Hnf4a*) and treated with vehicle or MG132. Staining performed with antibody to P1-HNF4α. Overlay with DAPI nuclear stain. (Scale bar 20µm).

**
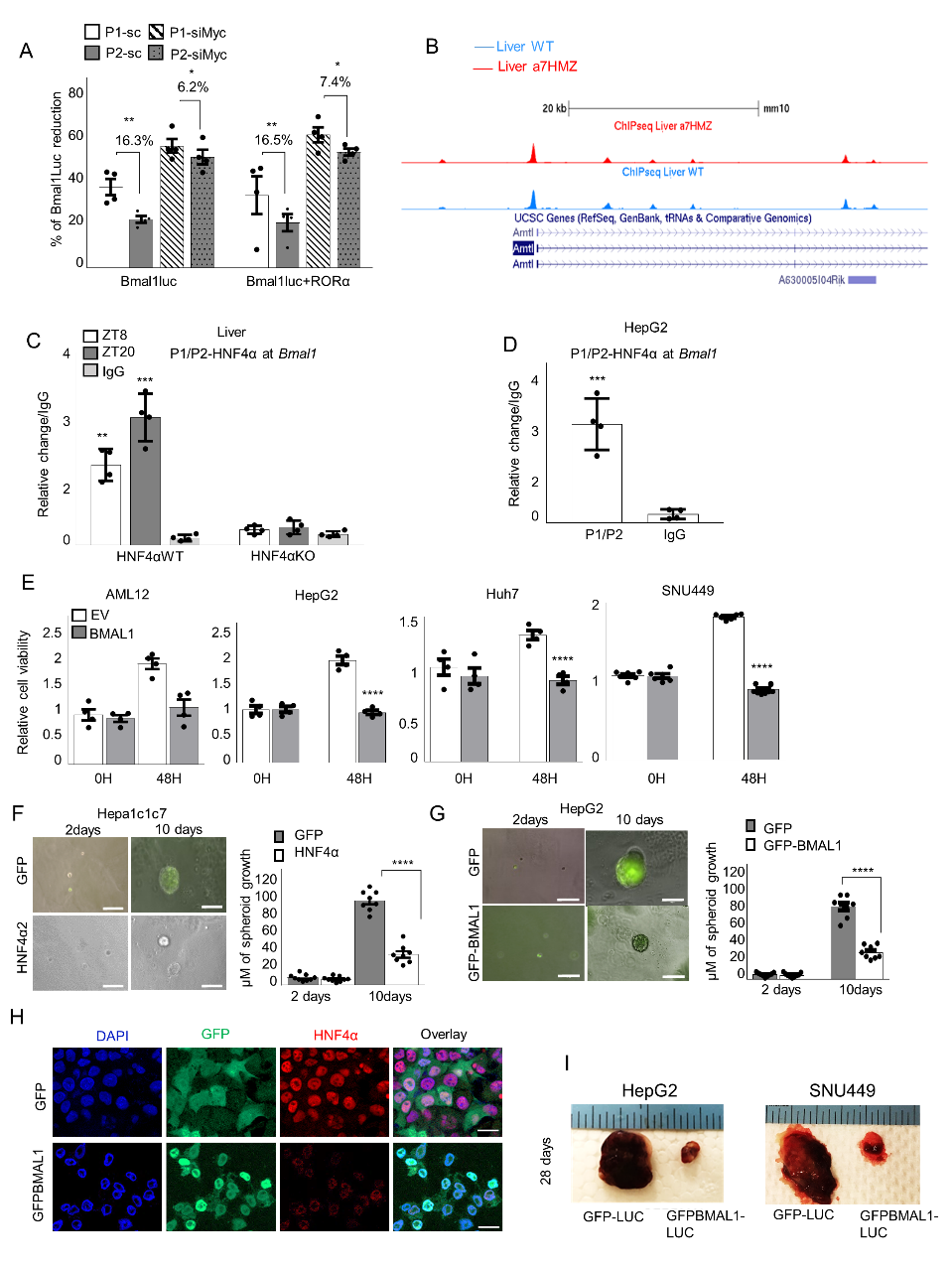
**

**Supplementary Figure 6 Co-Expression of BMAL1 and HNF4α in HCC Impairs Cell Proliferation** (A) Quantification of MYC-mediated repression of *Bmal1-luc* activity in the presence or absence of siRNA to the P1- or P2- HNF4α. (Corresponds to Figure 6A.) (B) ChIP-seq peaks for P1-HNF4α or P2-HNF4α in WT or α7HMZ livers at the *Bmal1* (*Arntl*) locus. (C-D) Chromatin immunoprecipitation of HNF4α followed by qPCR reveals P1/P2 isoform occupancy at an upstream *Bmal1* gene regulatory region in mouse WT and *Hnf4a* KO liver at two different *zeitgeber* times, ZT8 and ZT20 (left panel), and in the HepG2 cell line in unsynchronized conditions (right panel). Compared to IgG: *P<0.05, **P<0.01, ***P<0.001, ****P<0.0001, *One-way ANOVA* test, Dunnett’s multiple comparisons test, (N=5). (E) Fold change in forty-eight hour cell proliferation as measured by MTT assay of AML12, Huh7, and HepG2 cells following overexpression of *Bmal1.* *Two-way ANOVA*, Sidak’s multiple comparisons test, *P<0.03, **P<0.005, ***P<0.0005, ****P<0.0001, (N=6)*.* (F) 3D organoid growth in Matrigel at 2 and 10 days following plating of Hepa-1c1c7 cells transfected with *Gfp* empty vector or *Gfp-P1-Hnf4a* expression vector. Quantification of spheroid size at 2 and 10 days after plating, right panel. (G) 3D organoid growth in Matrigel at 2 and 10 days following plating of HepG2 cells transfected with *Gfp* empty vector or *Gfp-P1-Bmal1* expression vector. Quantification of spheroid size at 2 and 10 days after plating, right panel. *Two-way ANOVA*, Sidak’s multiple comparisons test, *P<0.03, **P<0.005, ***P<0.0005, ****P<0.0001, (N=9), (Scale bar 100µm). (H) Staining of HepG2 cells after transient transfection with *Gfp*-containing empty vector or *Gfp-Bmal1*. Staining was performed with antibodies to P1/P2-HNF4α and BMAL1. Counterstained with DAPI nuclear stain. (I) HepG2 and SNU449-derived tumors excised from the xenograft experiments. (Corresponds to Figure 6C.) Error bars=SEM.

**
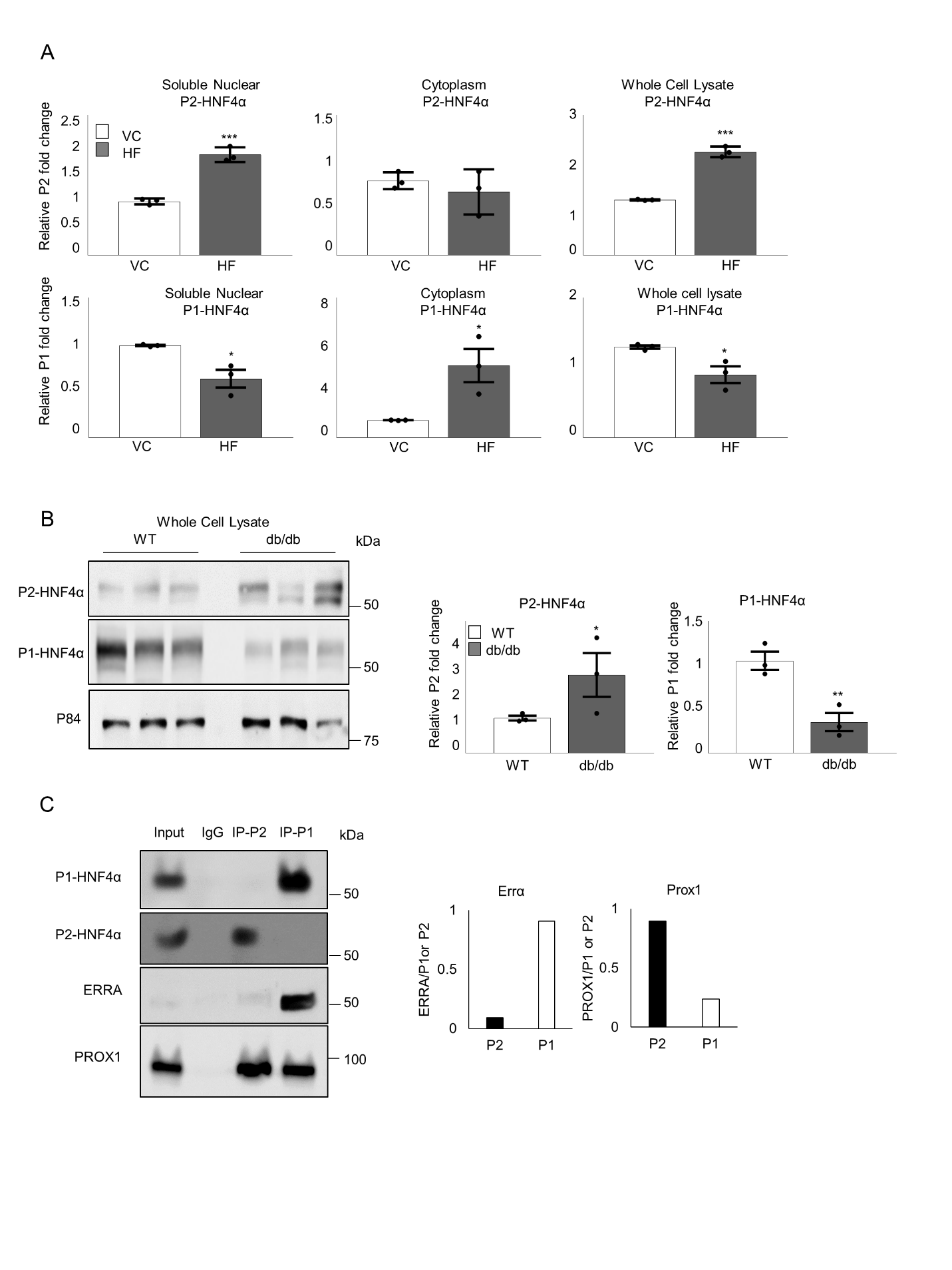
**

**Supplementary Figure 7 P2-HNF4a is induced in Fatty Liver** (A) Quantification of HNFα isoforms in the nuclear and cytoplasmic cell fractions as well as in whole cell lysates of mice made obese by prolonged HFD feeding. (B) Western blot analysis reveals P1-HNF4α, P2-HNF4α and P84 in livers from leptin-deficient mice (*db/db*). Western blot quantification (right panel). (C) Western blot reveals co-immunoprecipitation of ERRA and PROX1 with P1- HNF4α or P2-HNF4α. Quantification of ERRA and PROX1 normalized to the amount of HNF4α immunoprecipitated.

**
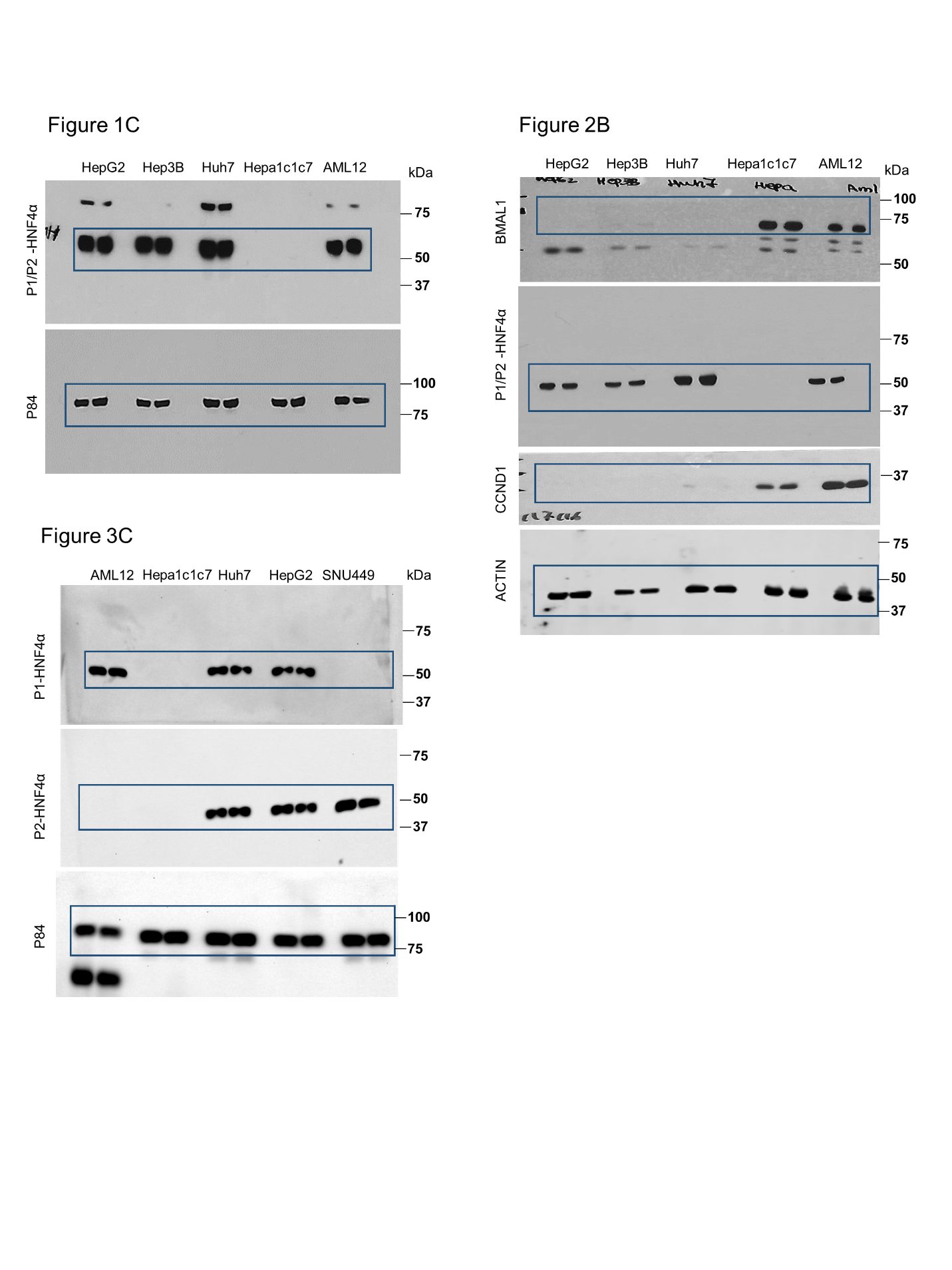
**

**Supplementary Figure 8**. Original western blots shown in figures 1C, 2B and 3C. Each figure corresponds to the western blots in the indicated figure number.


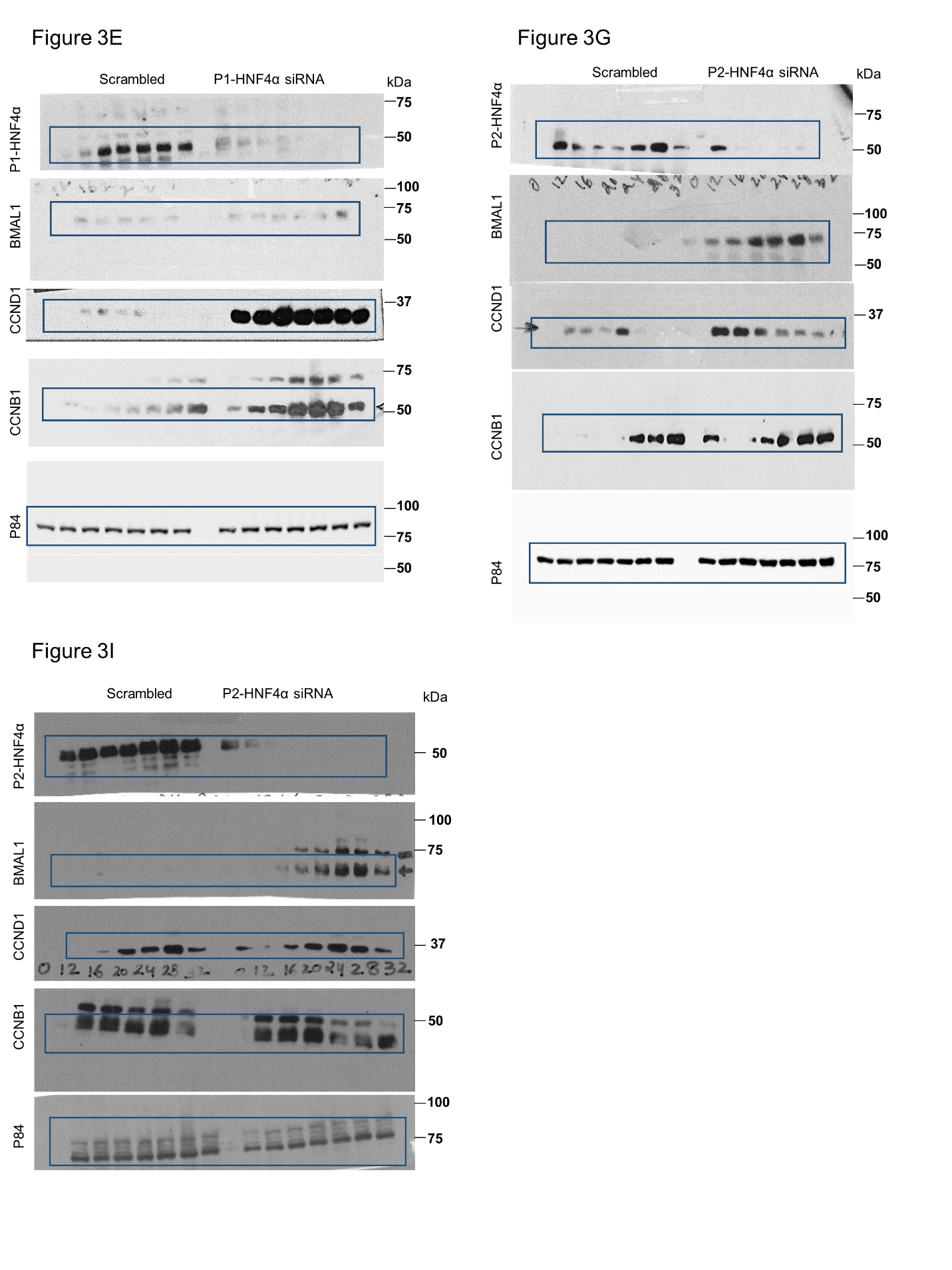


**Supplementary Figure 9**. Original western blots shown in figures 3E, 3G and 3I. Each figure corresponds to the western blots in the indicated figure number.


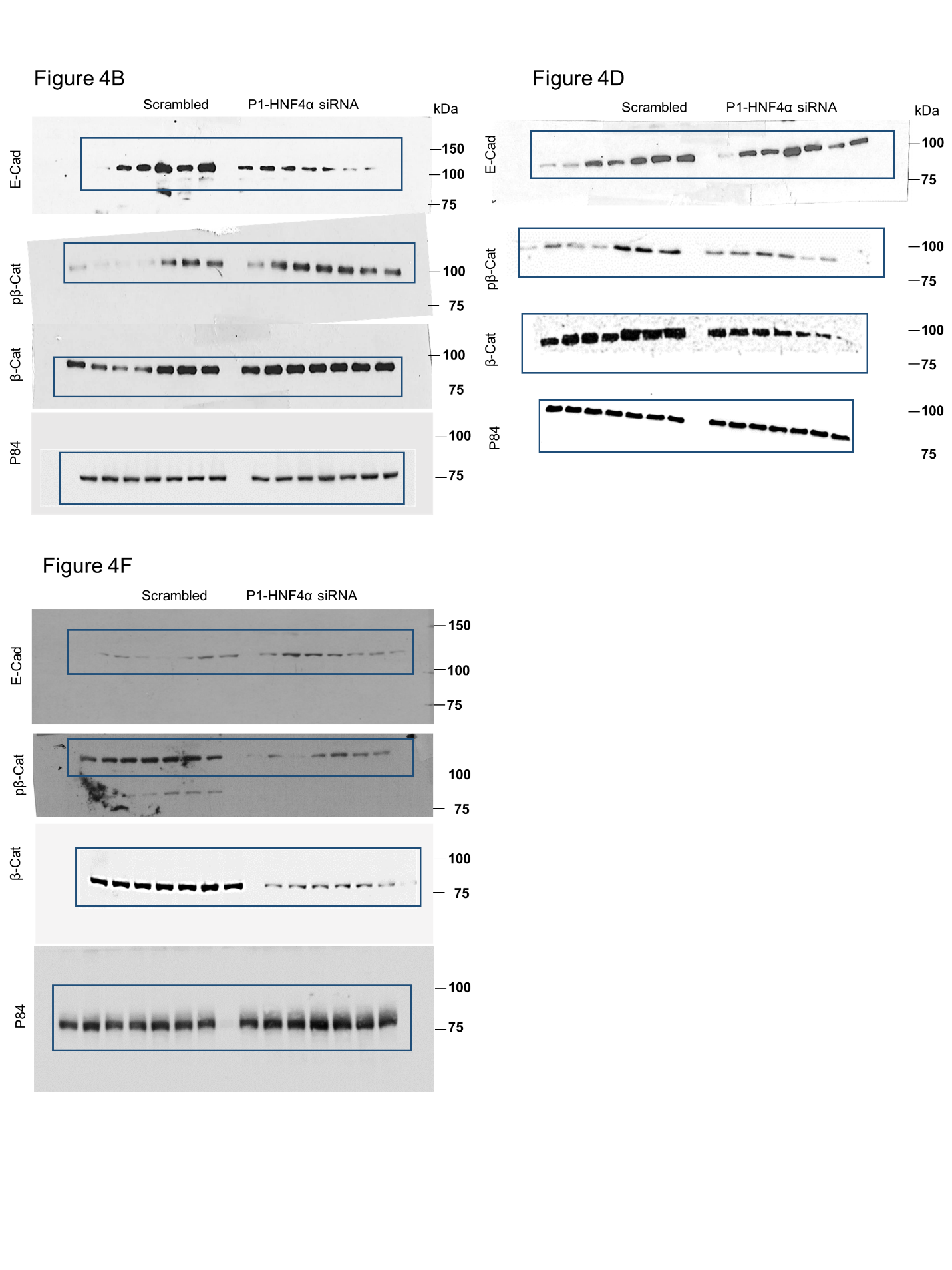


**Supplementary Figure 10**. Original western blots shown in figures 4B, 4D and 4F. Each figure corresponds to the western blots in the indicated figure number.

**
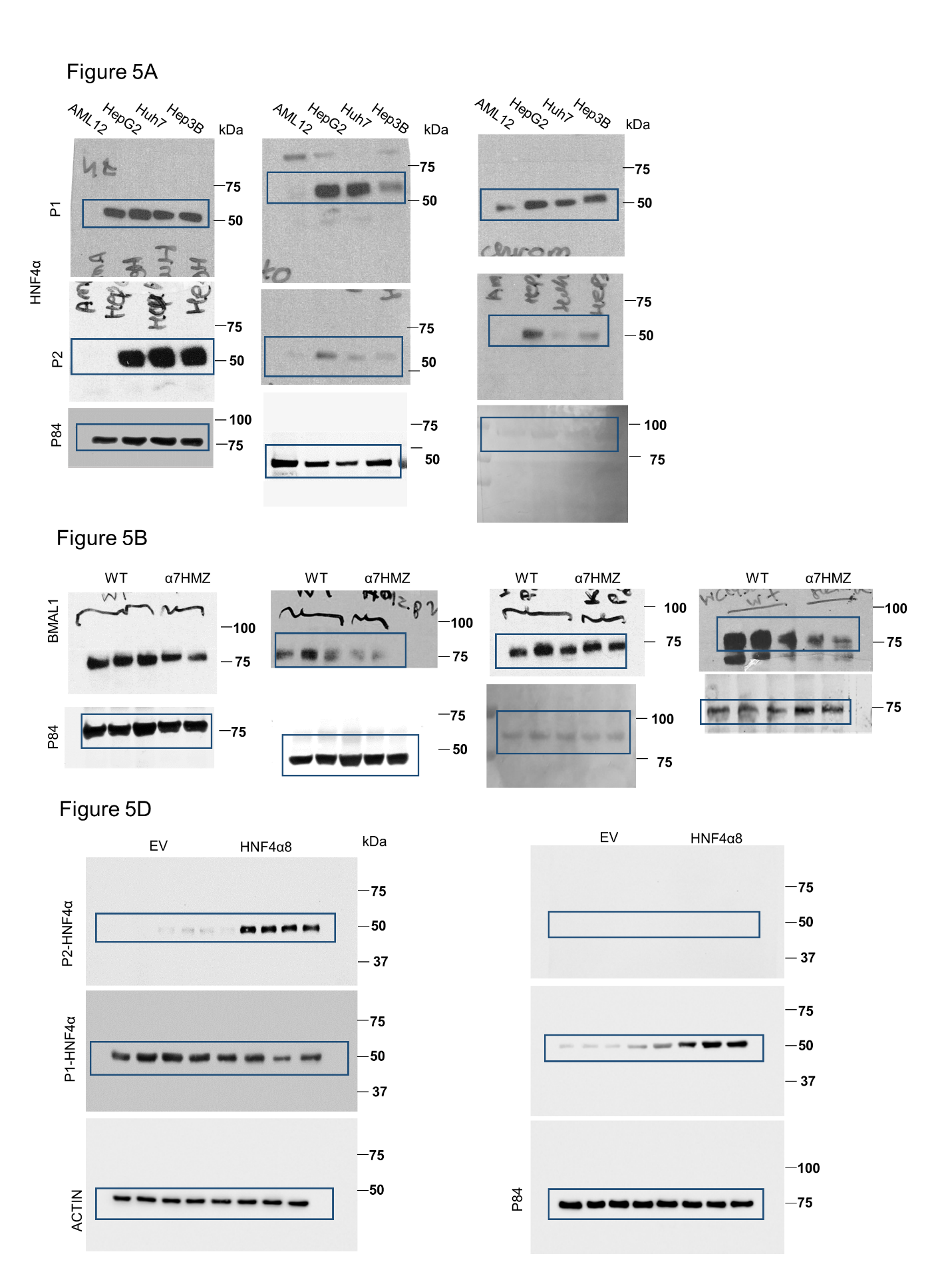
**

**Supplementary Figure 11**. Original western blots shown in figures 5A, 5B and 5D. Each figure corresponds to the western blots in the indicated figure number.

**
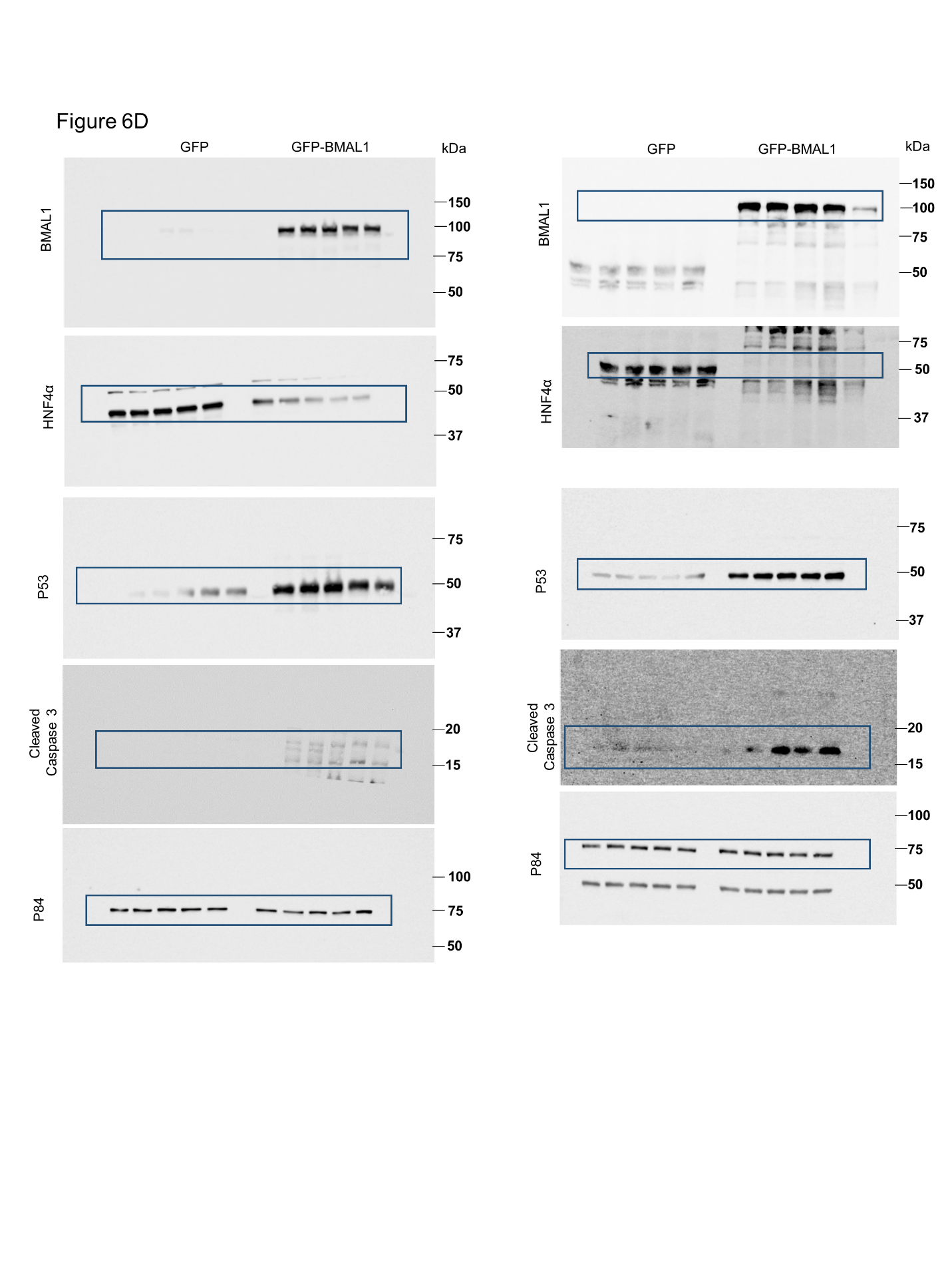
**

**Supplementary Figure 12**. Original western blots shown in figure 6D. Each figure corresponds to the western blots in the indicated figure number.


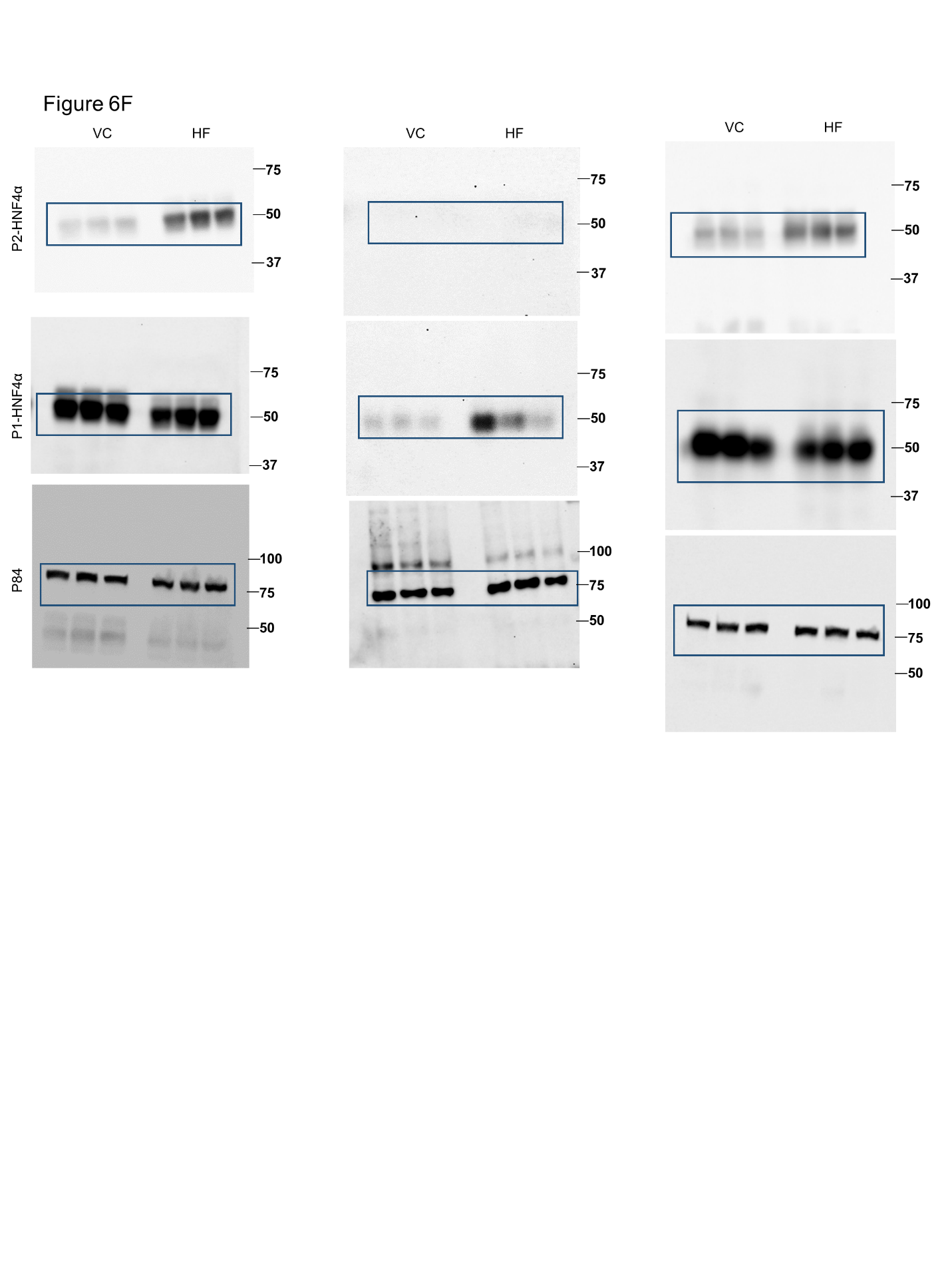


**Supplementary Figure 13**. Original western blots shown in figure 6F. Each figure corresponds to the western blots in the indicated figure number.

**
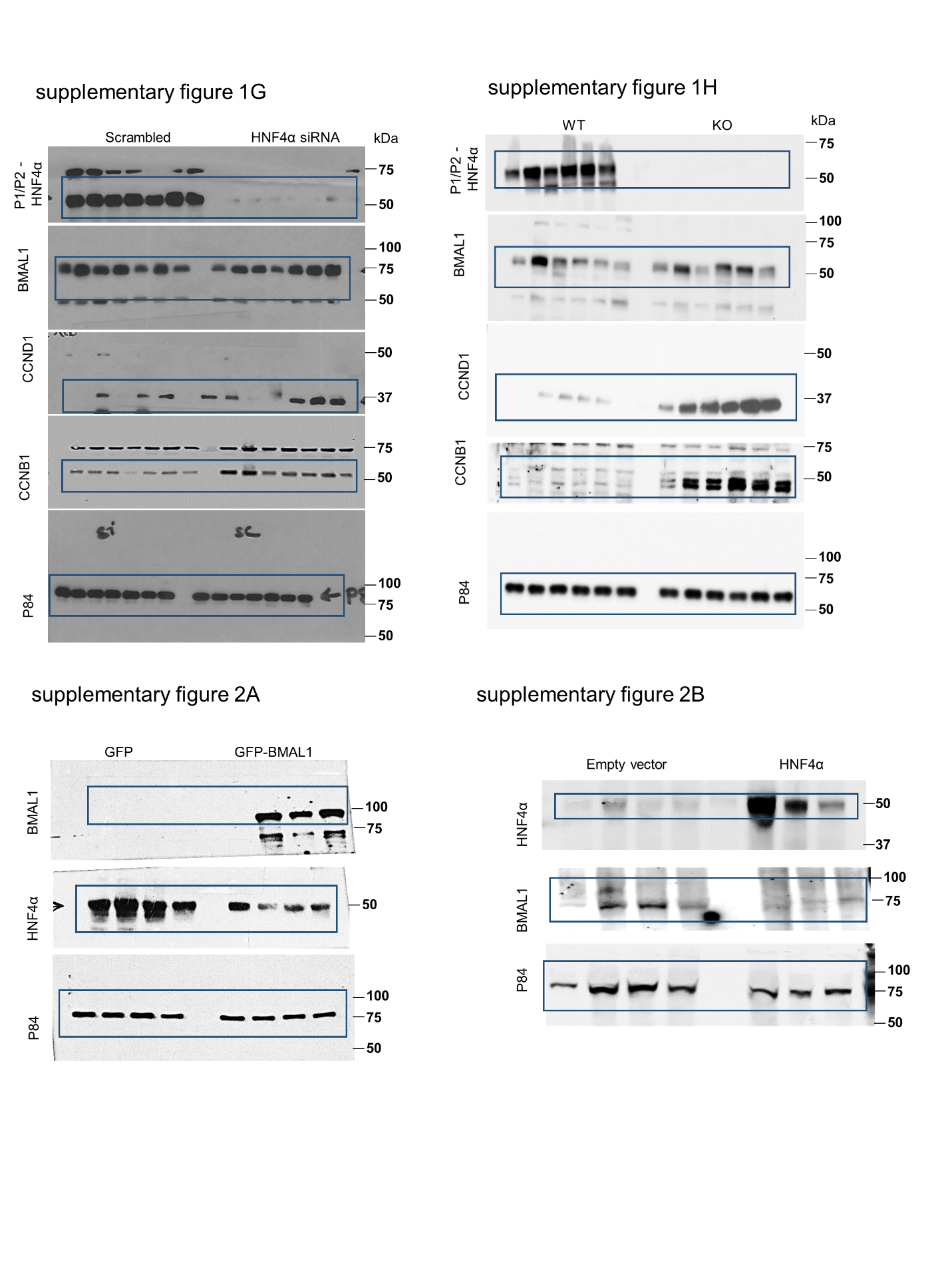
**

**Supplementary Figure 14**. Original western blots shown in supplementary figures 1G, 1H, 2A and 2B. Each figure corresponds to the western blots in the indicated figure number.

**
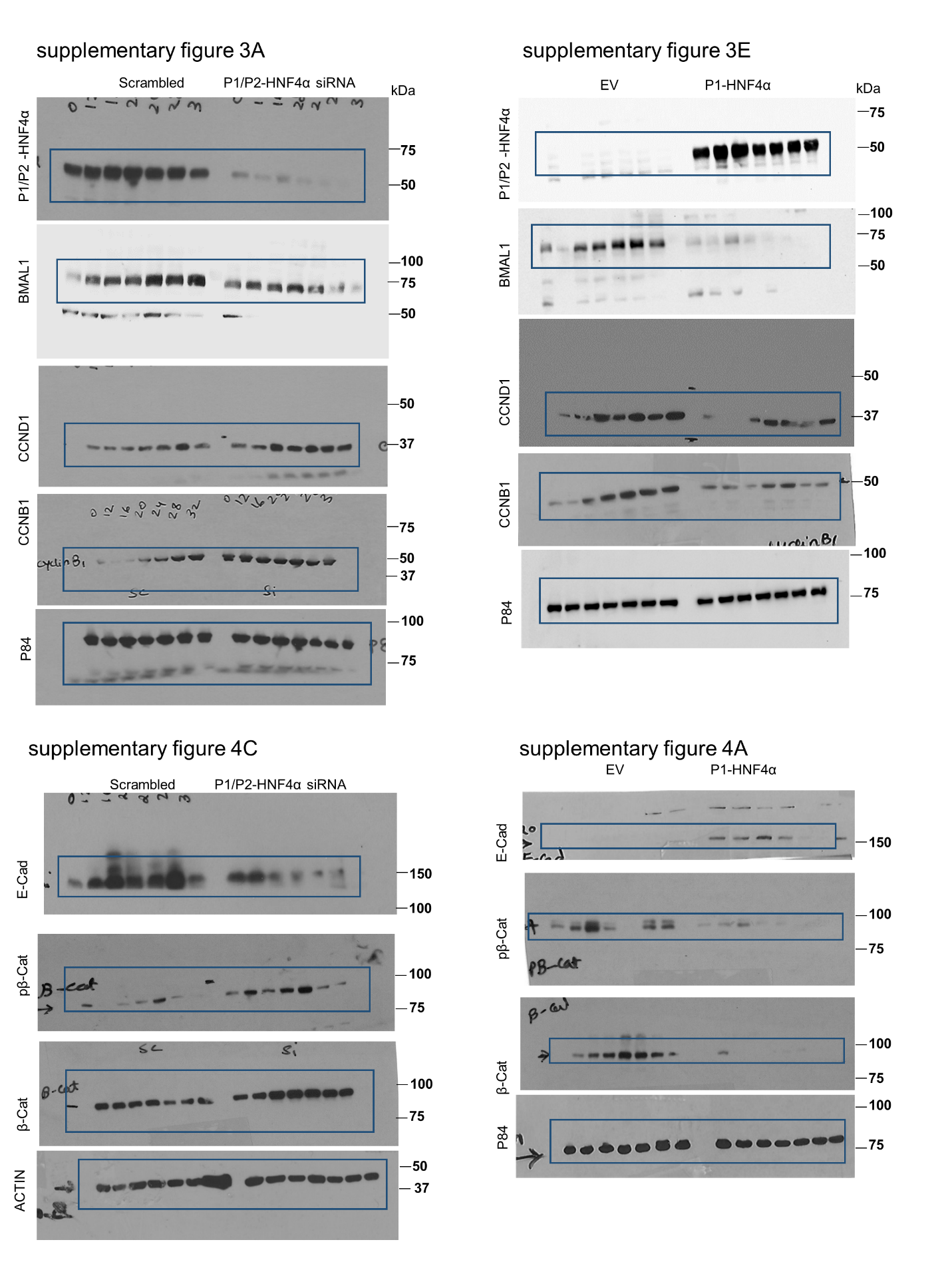
**

**Supplementary Figure 15**. Original western blots shown in supplementary figures 3A, 3E, 4A and 4C. Each figure corresponds to the western blots in the indicated figure number.

**
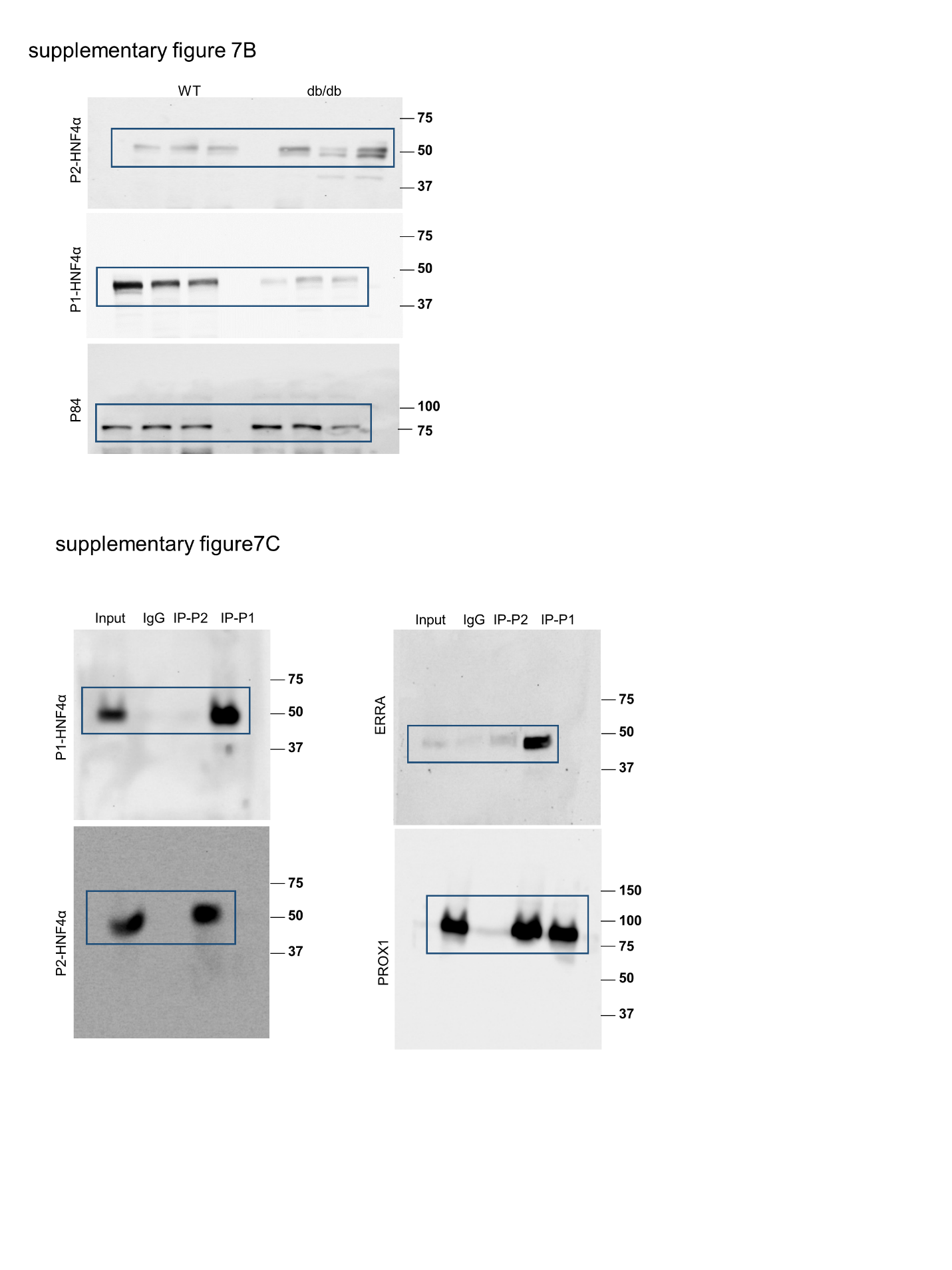
**

**Supplementary Figure 16**. Original western blots shown in supplementary figure 7B and C. Each figure corresponds to the western blots in the indicated figure number.

**Supplementary Methods**

**Supplementary Table 1: JTK results**

Figure 1H, S1E

| AML12- Scrambled | BH.Q | | | ADJ.P | | PER | LAG | | | AMP | | AML12-P1/P2 HNF4siRNA | | | BH.Q | | ADJ.P | | | PER | | | LAG | | | AMP |
| --- | --- | --- | --- | --- | --- | --- | --- | --- | --- | --- | --- | --- | --- | --- | --- | --- | --- | --- | --- | --- | --- | --- | --- | --- | --- | --- |
| *Dbp* | 0.0207547 | | | 0.0075472 | | 20 | 6 | | | 1.3764942 | | *Dbp* | | | 0.2290315 | | 0.1041052 | | | 20 | | | 10 | | | 1.371 |
| *Bmal1* | 1 | | | 0.7034617 | | 20 | 0 | | | 0.6481151 | | *Bmal1* | | | 0.13078 | | 0.0359403 | | | 20 | | | 6 | | | 0.965 |
| *Per2* | 0.0095059 | | | 0.0017284 | | 20 | 16 | | | 0.4831049 | | *Per2* | | | 0.1094882 | | 0.0199069 | | | 20 | | | 16 | | | 0.5042 |
| *Clock* | 1 | | | 0.9953562 | | 20 | 8 | | | 0.3225322 | | *Clock* | | | 0.13078 | | 0.0475564 | | | 20 | | | 8 | | | 0.4517 |
| *Ccnb1* | 0.463709 | | | 0.2107768 | | 20 | 14 | | | 0.1430682 | | *Ccnb1* | | | 0.2639699 | | 0.1679809 | | | 20 | | | 12 | | | 0.06 |
| *Myc* | 1 | | | 0.9953562 | | 24 | 14 | | | 0.2562881 | | *Myc* | | | 0.2898181 | | 0.2107768 | | | 20 | | | 10 | | | 0.2595 |
| *Ccnd1* | 1 | | | 1 | | 24 | 18 | | | 0.3742306 | | *Ccnd1* | | | 1 | | 0.9953562 | | | 20 | | | 4 | | | 0.7784 |
| *Snai1* | 1 | | | 1 | | 20 | 4 | | | 0.1432811 | | *Snai1* | | | 1 | | 1 | | | 20 | | | 4 | | | 0.2011 |
| *Snai2* | 0.0083053 | | | 0.000755 | | 24 | 20 | | | 0.3509877 | | *Snai2* | | | 0.5918753 | | 0.4842616 | | | 24 | | | 20 | | | 0.1535 |
| *Cdh1* | 0.0195439 | | | 0.0053301 | | 20 | 6 | | | 1.2029763 | | *Cdh1* | | | 0.0083053 | | 0.000755 | | | 24 | | | 6 | | | 0.7932 |
| P1/P2 *Hnf4a* | 1 | | | 0.5856348 | | 24 | 10 | | | 0.3817563 | | P1/P2 *Hnf4a* | | | 0.2639699 | | 0.1679809 | | | 24 | | | 6 | | | 0.0335 |
| Figure 1H,S1F |  | |  | | |  | |  | | |  | |  | | |  | | |  | | |  | | |  | |
| WTLiv | BH.Q | | | ADJ.P | | PER | LAG | | | AMP | | KOLiv | | | BH.Q | | ADJ.P | | | PER | | | LAG | | | AMP |
| *Ccnd1* | 0.0364961 | | | 0.0199069 | | 24 | 10 | | | 0.5157438 | | *Ccnd1* | | | 0.0070199 | | 0.0025527 | | | 24 | | | 12 | | | 5.6365 |
| *Ccnb1* | 0.7157759 | | | 0.5856348 | | 24 | 4 | | | 0.2007441 | | *Ccnb1* | | | 0.0166038 | | 0.0075472 | | | 20 | | | 0 | | | 5.9492 |
| *Bmal1* | 0.0001204 | | | 2.19E-05 | | 20 | 0 | | | 0.325374 | | *Bmal1* | | | 6.53E-05 | | 1.19E-05 | | | 24 | | | 0 | | | 0.5969 |
| *Dbp* | 1.31E-06 | | | 1.19E-07 | | 20 | 10 | | | 44.671303 | | *Dbp* | | | 1.66E-05 | | 1.51E-06 | | | 20 | | | 10 | | | 77.03 |
| *Myc* | 0.0102152 | | | 0.0037146 | | 24 | 10 | | | 0.4710401 | | *Myc* | | | 0.0979316 | | 0.0623201 | | | 24 | | | 12 | | | 2.0645 |
| *Cdh1* | 1 | | | 1 | | 20 | 2 | | | 0.1428277 | | *Cdh1* | | | 0.8597865 | | 0.7034617 | | | 24 | | | 0 | | | 0.2537 |
| *Snai1* | 1 | | | 1 | | 20 | 6 | | | 0.2542644 | | *Snai1* | | | 1 | | 1 | | | 20 | | | 10 | | | 0.1537 |
| *Snai2* | 0.0117263 | | | 0.0053301 | | 24 | 8 | | | 0.3351312 | | *Snai2* | | | 0.0979316 | | 0.0623201 | | | 24 | | | 6 | | | 0.3453 |
| *Per2* | 0.0002492 | | | 6.80E-05 | | 24 | 16 | | | 1.4082832 | | *Per2* | | | 0.0011259 | | 0.0003071 | | | 24 | | | 16 | | | 1.1203 |
| *Clock* | 0.5467886 | | | 0.3976645 | | 24 | 2 | | | 0.3401875 | | *Clock* | | | 0.4458122 | | 0.324227 | | | 20 | | | 0 | | | 0.2967 |
| P1/P2 *Hnf4a* | 0.0979316 | | | 0.0623201 | | 24 | 14 | | | 0.3128289 | | P1/P2 *Hnf4a* | | | 1 | | 1 | | | 24 | | | 12 | | | 0.0157 |
| Figure S3B,S4D |  | |  | | |  | |  | | |  | |  | | |  | | |  | | |  | | |  | |
| HepG2- *Scrambled* | BH.Q | | | ADJ.P | | PER | LAG | | | AMP | | HepG2- P1/P2 HNF4siRNA | | | BH.Q | | ADJ.P | | | PER | | | LAG | | | AMP |
| *DBP* | 0.0422084 | | | 0.0105521 | | 24 | 12 | | | 0.6540975 | | *DBP* | | | 0.0297171 | | 0.0037146 | | | 24 | | | 0 | | | 1.2436 |
| *CCND1* | 4.97E-05 | | | 6.21E-06 | | 24 | 10 | | | 0.2899138 | | *CCND1* | | | 1 | | 0.9953562 | | | 20 | | | 0 | | | 0.6037 |
| *CCNB1* | 1 | | | 1 | | 20 | 18 | | | 0.2801511 | | *CCNB1* | | | 1 | | 1 | | | 24 | | | 10 | | | 0.4412 |
| *SNAI1* | 1 | | | 1 | | 20 | 16 | | | 0.0753678 | | *SNAI1* | | | 1 | | 0.9953562 | | | 20 | | | 18 | | | 0.5303 |
| *SNAI2* | 1 | | | 1 | | 20 | 6 | | | 0.9279505 | | *SNAI2* | | | 1 | | 1 | | | 24 | | | 22 | | | 3.2578 |
| *CDH1* | 1 | | | 1 | | 24 | 10 | | | 0.6286297 | | *CDH1* | | | 1 | | 0.324227 | | | 24 | | | 4 | | | 0.1306 |
| *CTNNB1* | 1 | | | 1 | | 24 | 6 | | | 0.2375421 | | *CTNNB1* | | | 1 | | 0.8394588 | | | 24 | | | 6 | | | 0.1873 |
| P1/P2 *HNF4α* | 1 | | | 1 | | 24 | 0 | | | 0.0955732 | | P1/P2 *HNF4α* | | | 1 | | 1 | | | 20 | | | 16 | | | 0.0056 |
| Figure 3D,F,4A |  | |  | | |  | |  | | |  | |  | | |  | | |  | | |  | | |  | |
| HepG2- *Scrambled* | BH.Q | | | ADJ.P | | PER | LAG | | | AMP | | HepG2- P1 HNF4siRNA | | | BH.Q | | ADJ.P | | | PER | | | LAG | | | AMP |
| *DBP* | 0.215759 | | | 0.0809096 | | 20 | 6 | | | 1.0204545 | | *DBP* | | | 2.50E-05 | | 3.13E-06 | | | 20 | | | 8 | | | 0.9468 |
| *CCND1* | 0.0005437 | | | 6.80E-05 | | 20 | 4 | | | 0.757769 | | *CCND1* | | | 0.0002719 | | 6.80E-05 | | | 20 | | | 6 | | | 1.7877 |
| *CCNB1* | 1 | | | 1 | | 24 | 6 | | | 0.1554464 | | *CCNB1* | | | 0.215759 | | 0.0809096 | | | 24 | | | 8 | | | 0.3306 |
| *SNAI1* | 0.2655908 | | | 0.1327954 | | 24 | 12 | | | 0.2170108 | | *SNAI1* | | | 0.2687694 | | 0.1679809 | | | 24 | | | 22 | | | 0.7061 |
| *SNAI2* | 0.4198597 | | | 0.2624123 | | 20 | 6 | | | 0.9264292 | | *SNAI2* | | | 0.2655908 | | 0.1327954 | | | 24 | | | 6 | | | 0.9536 |
| *CDH1* | 1 | | | 1 | | 20 | 0 | | | 0.3678266 | | *CDH1* | | | 1 | | 1 | | | 20 | | | 16 | | | 0.1142 |
| *CTNNB1* | 0.0301887 | | | 0.0075472 | | 20 | 6 | | | 0.3505472 | | *CTNNB1* | | | 1 | | 1 | | | 24 | | | 12 | | | 0.1047 |
| P1 *HNF4α* | 1 | | | 1 | | 24 | 22 | | | 0.0752515 | | P1 *HNF4α* | | | 0.7808465 | | 0.5856348 | | | 24 | | | 6 | | | 0.0675 |
| P2 *HNF4α* | 0.01509 | | | 0.0007547 | | 20 | 4 | | | 1.1052 | | P2 *HNF4α* | | | 0.6484 | | 0.3242 | | | 20 | | | 4 | | | 1.11178 |
| Figure 3H,4C,3D |  | |  | | |  | |  | | |  | |  | | |  | | |  | | |  | | |  | |
| HepG2- *Scrambled* | BH.Q | | | ADJ.P | | PER | LAG | | | AMP | | HepG2- P2HNF4siRNA | | | BH.Q | | ADJ.P | | | PER | | | LAG | | | AMP |
| *DBP* | 0.215759 | | | 0.0809096 | | 20 | 6 | | | 1.0204545 | | *DBP* | | | 1 | | 1 | | | 20 | | | 2 | | | 1.453 |
| *CCND1* | 0.0005437 | | | 6.80E-05 | | 20 | 4 | | | 0.757769 | | *CCND1* | | | 0.9685233 | | 0.4842616 | | | 20 | | | 8 | | | 0.3415 |
| *CCNB1* | 1 | | | 1 | | 24 | 6 | | | 0.1554464 | | *CCNB1* | | | 0.8646054 | | 0.324227 | | | 24 | | | 6 | | | 0.1412 |
| *SNAI1* | 0.2655908 | | | 0.1327954 | | 24 | 12 | | | 0.2170108 | | *SNAI1* | | | 0.2492804 | | 0.0623201 | | | 20 | | | 14 | | | 0.0661 |
| *SNAI2* | 0.4198597 | | | 0.2624123 | | 20 | 6 | | | 0.9264292 | | *SNAI2* | | | 1 | | 0.8394588 | | | 24 | | | 0 | | | 0.2166 |
| *CDH1* | 1 | | | 1 | | 20 | 0 | | | 0.3678266 | | *CDH1* | | | 1 | | 1 | | | 20 | | | 6 | | | 0.5978 |
| *CTNNB1* | 0.0301887 | | | 0.0075472 | | 20 | 6 | | | 0.3505472 | | *CTNNB1* | | | 1 | | 1 | | | 20 | | | 6 | | | 0.3227 |
| P2 *HNF4α* | 0.00302 | | | 0.000755 | | 20 | 4 | | | 0.913689 | | P2 *HNF4α* | | | 0.1592556 | | 0.0199069 | | | 20 | | | 8 | | | 0.2557 |
| P1 *HNF4α* | 1 | | | 1 | | 20 | 8 | | | 0.1782 | | P1 *HNF4α* | | | 1 | | 1 | | | 20 | | | 10 | | | 0.24488 |
| Figure 3J,S3F,4E,S4F | |  | | |  |  | | |  | | |  | |  | | | |  | | |  | | |  | | |
| SNU449- *Scrambled* | BH.Q | | | ADJ.P | | PER | LAG | | | AMP | | SNU449- P2HNF4siRNA | | | BH.Q | | ADJ.P | | | PER | | | LAG | | | AMP |
| *DBP* | 0.0137169 | | | 0.008573 | | 20 | 10 | | | 0.3254219 | | *DBP* | | | 0.1854064 | | 0.0518037 | | | 28 | | | 0 | | | 0.6717 |
| *CCND1* | 0.0074385 | | | 0.0018719 | | 24 | 4 | | | 0.6203306 | | *CCND1* | | | 0.1854064 | | 0.0695274 | | | 28 | | | 10 | | | 0.4182 |
| *CCNB1* | 0.0137169 | | | 0.0071626 | | 20 | 16 | | | 0.4503803 | | *CCNB1* | | | 0.3977636 | | 0.2983227 | | | 24 | | | 8 | | | 0.1129 |
| *SNAI1* | 0.0064824 | | | 0.0008103 | | 28 | 10 | | | 0.2649716 | | *SNAI1* | | | 0.3293207 | | 0.2058255 | | | 28 | | | 18 | | | 0.363 |
| *SNAI2* | 0.0074385 | | | 0.0027894 | | 28 | 8 | | | 0.4477398 | | *SNAI2* | | | 1 | | 1 | | | 28 | | | 10 | | | 0.172 |
| *CDH1* | 0.1623795 | | | 0.1217846 | | 28 | 12 | | | 0.5699498 | | *CDH1* | | | 0.0183101 | | 0.0022888 | | | 20 | | | 14 | | | 1.2404 |
| *CTNNB1* | 1 | | | 1 | | 20 | 6 | | | 0.1960984 | | *CTNNB1* | | | 1 | | 1 | | | 28 | | | 10 | | | 0.1559 |
| P2 *HNF4α* | 1 | | | 1 | | 20 | 6 | | | 0.1178089 | | P2 *HNF4α* | | | 0.2435693 | | 0.1217846 | | | 20 | | | 10 | | | 0.0874 |
| Figure S3E,S4A |  | | |  | |  |  | | |  | |  | | |  | |  | | |  | | |  | | |  |
| Hep1c1c-EV | BH.Q | | | ADJ.P | | PER | LAG | | | AMP | | Hep1c1c-HFN4α2 | | | BH.Q | | ADJ.P | | | PER | | | LAG | | | AMP |
| *Dbp* | 0.0026426 | | | 0.0004861 | | 24 | 14 | | | 0.5490609 | | *Dbp* | | | 0.0665789 | | 0.0475564 | | | 24 | | | 10 | | | 0.3454 |
| *Ccnd1* | 1 | | | 1 | | 24 | 18 | | | 0.3742306 | | *Ccnd1* | | | 0.0628956 | | 0.0359403 | | | 20 | | | 6 | | | 1.3945 |
| *Ccnb1* | 0.4592215 | | | 0.2624123 | | 20 | 4 | | | 0.6159098 | | *Ccnb1* | | | 0.0013301 | | 0.00019 | | | 20 | | | 10 | | | 0.4226 |
| *Snai1* | 1 | | | 1 | | 20 | 4 | | | 0.143281 | | *Snai1* | | | 1 | | 1 | | | 20 | | | 4 | | | 0.2011 |
| *Snai2* | 0.0026426 | | | 0.000755 | | 24 | 20 | | | 0.3509873 | | *Snai2* | | | 0.5649719 | | 0.4842616 | | | 24 | | | 20 | | | 0.1535 |
| *Cdh1* | 0.012437 | | | 0.0053301 | | 20 | 6 | | | 1.202976 | | *Cdh1* | | | 0.0026426 | | 0.000755 | | | 24 | | | 6 | | | 0.7932 |
| *Myc* | 0.6779663 | | | 0.4842616 | | 20 | 16 | | | 0.6199137 | | *Myc* | | | 0.0464495 | | 0.0199069 | | | 24 | | | 2 | | | 0.3186 |
| FigureS1I,J |  | |  | | |  | |  | | |  | |  | | |  | | |  | | |  | | |  | |
| HepG2- Scrambled | BH.Q | | | ADJ.P | | PER | LAG | | | AMP | | HepG2- BMAL1siRNA | | | BH.Q | | ADJ.P | | | PER | | | LAG | | | AMP |
| *BMAL1* | 0.5547619 | | | 0.5547619 | | 0 | 0 | | | 0 | | *BMAL1* | | | 0.5547619 | | 0.5547619 | | | 0 | | | 0 | | | 0 |
| P1/P2 *HNF4α* | 0.0571429 | | | 0.0190476 | | 24 | 12 | | | 0.2471592 | | P1/P2 *HNF4α* | | | 0.5547619 | | 0.5547619 | | | 24 | | | 12 | | | 0.229 |
| *CCND1* | 0.3678571 | | | 0.2452381 | | 24 | 0 | | | 0.9887537 | | *CCND1* | | | 0.25 | | 0.0833333 | | | 24 | | | 18 | | | 0.223 |
| HepG2- P1/P2HNF4siRNA | BH.Q | | | ADJ.P | | PER | LAG | | | AMP | | HepG2- P2HNF4siRNA+ BMAL1siRNA | | | BH.Q | | ADJ.P | | | PER | | | LAG | | | AMP |
| *BMAL1* | 1 | | | 1 | | 24 | 0 | | | 0.5144854 | | *BMAL1* | | | 0.1285714 | | 0.0428571 | | | 24 | | | 0 | | | 0.1869 |
| P1/P2 *HNF4α* | 0.0571429 | | | 0.0190476 | | 24 | 12 | | | 0.2471592 | | P1/P2 *HNF4α* | | | 0.2214286 | | 0.147619 | | | 0 | | | 0 | | | 0 |
| *CCND1* | 0.3678571 | | | 0.2452381 | | 0 | 0 | | | 0 | | *CCND1* | | | 0.3785714 | | 0.3785714 | | | 0 | | | 0 | | | 0 |
| Figure S1D |  | |  | | |  | |  | | |  | |  | | |  | | |  | | |  | | |  | |
| Hepatocyte | BH.Q | | | ADJ.P | | PER | LAG | | | AMP | | Hep3B | | | BH.Q | | ADJ.P | | | PER | | | LAG | | | AMP |
| *Dbp* | 0.0199069 | | | 0.0199069 | | 20 | 8 | | | 1.8364853 | | *DBP* | | | 0.0037146 | | 0.0037146 | | | 20 | | | 6 | | | 1.0642 |
| Aml12 | BH.Q | | | ADJ.P | | PER | LAG | | | AMP | | HepG2 | | | BH.Q | | ADJ.P | | | PER | | | LAG | | | AMP |
| *Dbp* | 1 | | | 1 | | 20 | 6 | | | 3.0538496 | | *DBP* | | | 0.9953562 | | 0.9953562 | | | 20 | | | 8 | | | 0.2563 |
| Hepa1c1c | BH.Q | | | ADJ.P | | PER | LAG | | | AMP | |  | | |  | |  | | |  | | |  | | |  |
| *Dbp* | 0.0268891 | | | 0.0268891 | | 20 | 8 | | | 0.6235655 | |  | | |  | |  | | |  | | |  | | |  |

**Supplementary Table 2: Plasmid Resources**

| Plasmid | Source |
| --- | --- |
| pcDNA3.1 HNF4α2 | Gift from Dr Frances Sladek ^1^ ^1^ |
| pcDNA3.1 HNF4α8 | Gift from Dr Frances Sladek ^1^ |
| pcDNA3.1 flagBmal1 | Gift from Dr by Dr. Seung-Hee Yoo ^2^ |
| pcDNA3.1 flag RORα | Gift from Dr by Dr. Seung-Hee Yoo ^3^ |
| pcDNA3.1flag | Gift from Dr by Dr. Seung-Hee Yoo ^3^ |
| pEGFP-C1 | CLONTECH Cat#6086-1 |
| pGL3 Bmal-luc | Gift from Shin Yamazaki ^4^ |
| pLenti CMV Puro LUC(Transfer vector) | Eric Campeau (Addgene plasmid # 17477) ^5^ |
| pLenti CMV GFP Puro(Transfer vector) | Eric Campeau (Addgene plasmid # 17448) ^5^ |
| pMD2.G (Envelope vector) | Didier Trono (Addgene plasmid # 12259) (unpublished) |
| pRSV-Rev(Packaging vector ) | Didier Trono (Addgene plasmid # 12253) ^6^ |
| pMDLg/pRRE(Packaging vector ) | Didier Trono (Addgene plasmid # 12251) ^6^ |
| pGL3Luc(Transfer vector) | Joshua Mendell (Addgene plasmid # 64784) ^7^ |
| pLenti CMV GFP Bmal1(Transfer vector) | This Study |
| pLenti CMV Hnf4α(Transfer vector) | This Study |
| pEGFPBmal1 | This Study |
| pEGFP Hnf4α | This Study |
| pEGFP Hnf4α8 | This Study |
| PCDNA3.1 lacZ | Invitrogen |

**Supplementary Table 3: siRNA**

| Name | Sequences(5`to 3`) |
| --- | --- |
| Human P1/P2-HNF4α Sense | UGUGCAGGUGUUGACGAUGdTdT |
| Human P1/P2-HNF4α Antisense | CAUCGUCAACACCUGCACAdTdT |
| Human P1-HNF4α Sense | UUGAGAAUGUGCAGGUGUUUUdTdT |
| Human P1-HNF4α Antisense | UUAACUCUUACACGUCCACA AdTdT |
| Human P2-HNF4α Sense | GUGGAGAGUUCUUACGACAUUdTdT |
| Human P2-HNF4α Antisense | UUCACCUCUCAAGAAUGCUGUdTdT |
| Mouse P1/P2-HNF4α Sense | CGCUUGAGGAAGACCUACUdTdT |
| Mouse P1/P2-HNF4αAntisense | UCAUCCAGAAGGAGUUCGC dTdT |
| Mouse siMyc | THERMOFISHER Cat#AM16708 |
| Mouse siBmal1 | THERMOFISHER Cat#4390771 |
|  |  |

| Name | Sequences(5`to 3`) |
| --- | --- |
| ALB F | TTTGCAGATGTCAGTGAAAGAGA |
| ALB R | TGGGGAGGCTATAGAAAATAAGG |
| WPRE F | GTCCTTTCCATGGCTGCTC |
| WPRE R | CCGAAGGGACGTAGCAGA |

**Supplementary Table 4: Titration Primers**

**Supplementary Table 5: qPCR Primers**

| Name | Sequences(5`to 3`) Mouse | Sequences(5`to 3`) Human |
| --- | --- | --- |
| Bmal1 F | GCAGTGCCACTGACTACCAAGA | TTAGCCAACGTCCTGGAAGG |
| Bmal1 R | TCCTGGACATTGCATTGCAT | CCTTCTCCAGAGGGCAGCAT |
| CMyc F | ACCACCAGCAGCGACTCT | AACACAAACTTGAACAGCTAC |
| CMyc F | GCTGTGAGGAGGTTTGCTGT | ATTTGAGGCAGTTTACATTATGG |
| Clock F | ACCACAGCAACAGCAACAAC | TTCTCTTCTTCCGTCCACCC |
| Clock R | GGCTGCTGAACTGAAGGAAG | TTCTCTTCTTCCGTCCACCC |
| CyclinD1 F | TGCTACCGCACAACGCA | AAGGCGGAGGAGACCTGCGCG |
| CyclinD1 R | TCAATCTGTTCCTGGCAGGC | ATCGTGCGGCATTGCGGC |
| Cyclin B1 F | GGAAATTCTTGACAACGGTG | AATAAGGCGAAGATCAACATGGC |
| Cyclin B1 R | TGCCTTTGTCACGGCCTTAG | TTTGTTACCAATGTCCCCAAGAG |
| DBP F | AATGACCTTTGAACCTGATCCCGCT | CGTGGAGGTGTTGATGACCTT |
| DBP R | GCTCCAGTACTTCTCATCCTTCTGT | CGATGTCTTCGAGGGTCAAAG |
| P1/P2 HNF4α F | ACCAAGAGGTCCATGGTGTTT | CCTACCTCAAAGCCATCAT |
| P1/P2 HNF4α R | GTGCCGAGGGACGATGTAG | ATGTAGTCCTCCAAGCTCAC |
| P1HNF4α F | CATGGATATGGCCGACTACAG | GTCGACATGGACATGGCCGACTACAGTG |
| P1HNF4α R | GCCCGAATGTCGCCATTGATCCCAGAGA | GCCCGAATGTCGCCGTTGATCCCGGAGACG |
| P2HNF4α F | CATGGTCAGTGTGAACGCGCCCCTCGG | GCCATGGTCAGCGTGAACGCGCCCCTCGG |
| P2HNF4α R | GCCCGAATGTCGCCATTGATCCCAGAGA | GCCCGAATGTCGCCGTTGATCCCGGAGACG |
| Per2 F | CGCCTAGAATCCCTCCTGAGA | GGGTGCGCTCGTTTGAACT |
| Per2 R | CCACCGGCCTGTAGGATCT | GGAACGAAGCTTTCGGACCTC |
| E-cadherin F | CAAAGTGACGCTGAAGTCCA | GTCAGTTCAGACTCCAGCCC |
| E-cadherin R | TGATGACACGGCATGAGAAT | AAATTCACTCTGCCCAGGACG |
| Snail1 F | CACCCTCATCTGGGACTCTC | ACCACTATGCCGCGCTCTT |
| Snail1 R | CTTCACATCCGAGTGGGTTT | GGTCGTAGGGCTGCTGGAA |
| Snail2 F | GCGAACTGGACACACACACAGTTAT | TGTTGCAGTGAGGGCAAGAA |
| Snail2 R | CCCCAGTGTGAGTTCTAATGTGTCC | GACCCTGGTTGCTTCAAGGA |
| *β*-catenin | ACAAACTGTTTTGAAAATCCA | AAGACATCACTGAGCCTG CCAT |
| *β*-catenin | CGAGTCATTGCATACTGTCC | CGATTTGCGGG CAAAGG GCAA |
| 18s F | CGCCGCTAGAGGTGAAATTC | TGACTCTAGATAACCTCGGG |
| 18S R | CGAACCTCCGACTTTCGTTCT | GACTCATTCCAATTACAGGG |

**Supplementary Table 6: Luciferase Assay Buffers**

| Luciferase assay Buffer | Composition |
| --- | --- |
| Luciferase Lysis buffer | 25m M Tris (pH 7.8), 2 mM EDTA, 10% glycerol,1% Triton X-100, 1mM DTT |
| Luciferase React Buffer | 20mM Tris Phosphate (pH7.8), 1mM MgCl_2_, 3mM MgSO_4_,0.2M EDTA, 60mM DTT, 0.5mM Luciferin, 0.1 mM ATP, 1mM CoA |
| Z buffer | 60mM Na_2_HPO_4_, 40mM NaH_2_PO_4_, 10mM Kcl, 1Mm MgSO4,50 mM beta-Mercaptoethanol |

**Supplementary Table 7: Protein Extraction Buffers**

| Buffer | Composition |
| --- | --- |
| RIPA | 50 mm Tris-HCl (pH 8.0),150 mM NaCl, EDTA 5mM, MgCl_2_ 15mM protease inhibitors ,and NP-40 1%, 1 mm PMSF, 1Mm NaF , 400mM NAM and 3.3 Mm TSA |
| Buffer A(BA) | 10 mM HEPES (pH 7.6), 25 mM KCL, 1 mM EDTA, 1 mM EGTA, 3%triton, 1mM DTT, protease inhibitors, , 1mM PMSF,1Mm NaF , 400mM NAM and 3.3 Mm TSA |
| Low Salt Buffer (LSB) | 10 mM HEPES (pH 7.6), 25 mM KCL, 0.1 mM EDTA,1 mM EGTA, 20% glycerol, 1 mM DTT ,protease inhibitors, 1mM PMSF,1Mm NaF , 400mM NAM and 3.3 Mm TSA |
| High Salt Buffer (HSB) | 10 mM HEPES (pH 7.6), 500 mM KCL, 0.1 mM EDTA,1 mM EGTA, 20% glycerol, 1 mM DTT , protease inhibitors, 1mM PMSF,1Mm NaF , 400mM NAM and 3.3 Mm TSA |

**Supplementary Table 8: Antibodies**

| Name | Catalog #, Company and working dilution for Western blotting (WB) or immunofluorescence (IF) |
| --- | --- |
| P1/P2-HNF4α | #PP-H1415-00 R&D Systems  WB (1:5000)- IF (1:500) |
| P1-HNF4α | #PP-K9218-00 R&D Systems  WB (1:2000)- IF (1:200) |
| P2-HNF4α | #PP-H6939-00 R&D Systems  WB (1:1000)- IF (1:100) |
| BMAL1 | #93806 Abcam  WB (1:1000) |
| BMAL1 | #NB100-129 [Novus Biologicals](https://www.googleadservices.com/pagead/aclk?sa=L&ai=DChcSEwjS0tOMzK_WAhUGgWkKHdD1DD8YABAAGgJpcQ&ohost=www.google.com&cid=CAASE-RoXQZW70jBsUUEYUG82GqIcQ4&sig=AOD64_0seqauInza9fq9njnsRcEFp89Zbw&q=&ved=0ahUKEwjl6cuMzK_WAhVD0GMKHf6HAMkQ0QwIJA&adurl=)  IF (1:100) |
| β-Catenin | #8 480 Cell Signaling  WB (1:1000) |
| Phospho-β-Catenin (Ser552) | #9566 Cell Signaling  WB (1:1000) |
| ERRα | #13826 Cell Signaling  WB (1:1000) |
| Prox1 | #07-537 Millipore  WB (1:1000) |
| E-Cadherin | #610181 BD Transduction Laboratories  WB (1:1000) |
| CCND1 | #EPR2241 Abcam  WB (1:10,000) |
| CCNB1 | #SC-245 Santa Cruz biotechnology  WB (1:200) |
| H3 | # GTX122181 GeneTex  WB (1:1000) |
| P84 | # GTX70220 GeneTex  WB (1:1000) |
| ACTIN | # A5441 Sigma  WB (1:5000) |
| TUBULIN | #ab6046 Abcam  WB (1:1000)- IF (1:400) |
| Anti-Mouse CF^™^488A antibody | #SAB4600388  Sigma  IF (1:500) |
| Anti-Rabbit IgG (H+L) Antibody Alexa Fluor 555 | #A-21430 Thermo Fisher  IF (1:500) |
| Anti-Rabbit IgG (H+L) Antibody Alexa Fluor 647 | #A-21235 Thermo Fisher  IF (1:500) |
| Anti-Mouse IgG (H+L) Antibody, Alexa Fluor 555 | **#**A-31570 Thermo Fisher  IF (1:500) |
| Anti-Mouse IRDYe 800CW | #926-32212 LICOR  WB (1:15,000) |
| Anti-Rabbit IRDYe 800CW | #925-32211 LICOR  WB (1:15,000) |
| Anti-Mouse-HRP | #1706516 BioRad  WB (1:10,000) |
| Anti-Rabbit-HRP | #1791019 BioRad  WB (1:10,000) |

**Supplementary Table 9: Construction Primers**

| Name | Sequences(5` to 3`) |
| --- | --- |
| SalI HNF4α-F | GAATCGGTCGACATGGATTACAAAGACGATGAC |
| SalI Bmal1-F | GAATCGGTCGACATGGCGGACCAGAGAATGGAC |
| XbaI-HNF4F | GAATCGTCTAGAATGGATTACAAAGACGATGAC |
| XbaI-Bmal1-F | GAATCGTCTAGATGGCGGACCAGAGAATGGAC |
| BamH1HNF4α-R | CGATTCGGATCCCTAGATGGCTTCCTGCTTGGT |
| BamH1Bmal1-R | CGATTCGGATCCCTACAGCGGCCATGGCAAGTC |
| SalI HNF4α8-F | GAATCGGTCGAC ATGGTCAGCGTGAACGCGCC |
| XbaI HNF4α8-F | CGATTCTCTAGA CTAGATAACTTCCTGCTTGGTG |

**Supplementary Table 10: Key Resources**

| REAGENT or RESOURCE | SOURCE | IDENTIFIER |
| --- | --- | --- |
| **Antibodies** | | |
| Antibodies used for immunoblotting , immunostaining and ChIP | See Table S8 | See [Table S8](http://www.sciencedirect.com.ezproxyhost.library.tmc.edu/science/article/pii/S0092867417308723#mmc1) |
| **Chemicals** | | |
| Protease inhibitors | ROCHE | Cat#11697498001 |
| NAM (nicotinamide) | SIGMA ALDRICH | Cat#N0636 |
| Trichostatin A | SIGMA ALDRICH | Cat# T8552 |
| Dynabeads Protein G | INVITROGEN | Cat#10003D |
| DSG (disuccinimidyl glutarate) | THERMOFISHER | Cat#2053 |
| BCA | PIERCE | Cat#23225 |
| Puromycin | FISHER SCIENTIFIC | Cat#NC9138068 |
| Clarity Chemiluminescence Reagent | BIORAD | Cat#170-5061 |
| VivoGlo Luciferin | PROMEGA | Cat#P1042 |
| CellTiter 96(MTT) | PROMEGA | Cat#G4000 |
| Proteinase K | LIFE TECHNOLOGIES | Cat#25530049 |
| TRIzol reagent | LIFE TECHNOLOGIES | Cat#15596018 |
| Insulin Solution | SIGMA ALDRICH | Cat#19278 |
| Sodium Selenite | SIGMA ALDRICH | Cat#S5261 |
| Transferrin | SIGMA ALDRICH | Cat# 11096-37 |
| Polybrene | EMD MILLIPORE | Cat#TR-1003 |
| Crystal violet | FISHER SCIENTIFIC | Cat#S25274B |
| Tamoxifen | SIGMA ALDRICH | Cat#T5648 |
| Dexamethasone | SIGMA ALDRICH | Cat#D8893 |
| SsoAdvanced Universal SYBR Green | BIORAD | Cat#4385618 |
| iScript cDNA reverse transcription kit | BIORAD | Cat#1708891 |
| Lenti-X™ Concentrator | CLONTECH | Cat#631231 |
| penicillin /streptomycin Solusion | GIBCO | Cat#1514022 |
| EMEM | ATCC | Cat#302003 |
| DMEM | GIBCO | Cat#12491-015 |
| MEM-alpha | GIBCO | Cat#12561-056 |
| DMEM/F12 | GIBCO | Cat#12634-010 |
| Opti-MEM | GIBCO | Cat#31985-070 |
| FBS | GENCLONE | Cat#25-514 |
| Horse Serum | GIBCO | Cat#26050088 |
| BamH1 | PROMEGA | Cat#R6021 |
| Xba1 | PROMEGA | Cat# R6181 |
| SalI | PROMEGA | Cat#R6051 |
| Go Taq | PROMEGA | Cat#M7806 |
| CIP | NEB | Cat#M0290S |
| Quick Ligation Kit | NEB | Cat#M2200s |
| Matrigel | CORNING | Cat#354234 |
| DAPI | LIFE TECHNOLOGIES | Cat#D1306 |
| MG132 | SIGMA ALDRICH | Cat#474791 |
| **Experimental Models: Cell Lines** |  |  |
| Hep 3B | ATCC | Cat#HB8064 |
| Hepa1c1c7 | ATCC | Cat#CRL2026 |
| Hep G2 | ATCC | Cat#HB8065 |
| Huh7 | ATCC | Cat#HB8561 |
| SNU449 | ATCC | Cat#CRL2234 |
| AML12 | ATCC | Cat#CRL2254 |
| HEK293-T LentiX | CLONTECH | Cat#63218 |
| **TMC Slides** |  |  |
| TMC | Usbiomax | [BS03013a](https://www.biomax.us/tissue-arrays/Liver/BS03013a) |
| TMC | Usbiomax | OD-CT-DgLiv02-001 |
| TMC | Usbiomax | OD-CT-DgLiv02-002 |
| TMC | Usbiomax | OD-CT-DgLiv02-003 |
| TMC | Usbiomax | OD-CT-DgLiv02-004 |
| TMC | Usbiomax | OD-CT-DgLiv02-005 |
| **Mouse Models** |  |  |
| NSG | Jackson lab | Cat#005557 |
| *Hnf4aF/F*;*AlbERT2cre* | Generated from gifts from Frank Gonzalez ( *Hnf4aF/F* ) Daniel Metzger, and Pierre Chambon (*AlbERT2cre)* | See Animal Section |
| α7HMZ |  | See Animal Section |
| **Plasmids** | N/A | See [Table S2](http://www.sciencedirect.com.ezproxyhost.library.tmc.edu/science/article/pii/S0092867417308723#mmc1) |
| Lipofectamine 2000 | THERMOFISHER | Cat#11668019 |
| Lipofectamine RNAi MAX | THERMOFISHER | Cat#13778075 |
| **Oligonucleotide** |  |  |
| Primers used for Cloning ,QPCR ,Lenti Virus titration and ChIP | Eurofins Genomics | See [Tables S1,](http://www.sciencedirect.com.ezproxyhost.library.tmc.edu/science/article/pii/S0092867417308723#mmc1)S4,S6 and S9 |
| **SiRNA targeting sequence** | Eurofins Genomics | See [Table S3](http://www.sciencedirect.com.ezproxyhost.library.tmc.edu/science/article/pii/S0092867417308723#mmc1) |
| **Software** |  |  |
| Prism 7 | Graph Pad |  |
| Fiji(Image J) | National Institutes Of Health, Usa |  |
| Zen | Zeiss | https://www.zeiss.com/microscopy/us/products/microscope-software/zen-lite.html |

**Supplementary References**

1 Vuong, L. M. *et al.* Differential Effects of Hepatocyte Nuclear Factor 4alpha Isoforms on Tumor Growth and T-Cell Factor 4/AP-1 Interactions in Human Colorectal Cancer Cells. *Mol Cell Biol* **35**, 3471-3490, doi:10.1128/MCB.00030-15 (2015).

2 Jeong, K. *et al.* Dual attenuation of proteasomal and autophagic BMAL1 degradation in Clock Delta19/+ mice contributes to improved glucose homeostasis. *Sci Rep* **5**, 12801, doi:10.1038/srep12801 (2015).

3 He, B. *et al.* The Small Molecule Nobiletin Targets the Molecular Oscillator to Enhance Circadian Rhythms and Protect against Metabolic Syndrome. *Cell Metab* **23**, 610-621, doi:10.1016/j.cmet.2016.03.007 (2016).

4 Yeom, M., Pendergast, J. S., Ohmiya, Y. & Yamazaki, S. Circadian-independent cell mitosis in immortalized fibroblasts. *Proc Natl Acad Sci U S A* **107**, 9665-9670, doi:10.1073/pnas.0914078107 (2010).

5 Campeau, E. *et al.* A versatile viral system for expression and depletion of proteins in mammalian cells. *PLoS One* **4**, e6529, doi:10.1371/journal.pone.0006529 (2009).

6 Dull, T. *et al.* A third-generation lentivirus vector with a conditional packaging system. *J Virol* **72**, 8463-8471 (1998).

7 Chang, T. C. *et al.* Transactivation of miR-34a by p53 broadly influences gene expression and promotes apoptosis. *Mol Cell* **26**, 745-752, doi:10.1016/j.molcel.2007.05.010 (2007).
